# Supplementary material for: Factors affecting enrollment status of households for community based health insurance in a resource-limited peripheral area in Southern Ethiopia. Mixed method
Source: PLoS One. 2021 Jan 25;16(1):e0245952. doi: 10.1371/journal.pone.0245952 (PMC7833211; doi:10.1371/journal.pone.0245952)
Supplement: S1 File — (DOCX) [file pone.0245952.s001.docx]

# **Annex I. Questionnaire**

Good morning/after noon, my name is______________________. I’m data collector of a research conducting on community based health insurance by Arba Minch University research team. The purpose of the study is to know the enrollment status of the households for community based health insurance and factors that enhance/hinder the community to enroll community based health insurance. To improve community based health insurance; your responses are very important. You are recruited for the interview by systematic random sampling method. This questionnaire consumes only 40 minutes. Any information you will provide as part of this interview will be held strictly confidential and your participation is voluntary. You don’t earn any benefit/incentive for participating in this study.

You can discontinue the interview if you feel uncomfortable at any time during the interview.

Are you volunteer to participate in this study? Agree Disagree

Questionnaire code _______

Participant signature___________________________

Data collector Name and signature_____________________

Contact Address of the principal Investigator: +251913976776, [mustesami02@gmail.com](mailto:mustesami02@gmail.com)

Sponsored by Arba Minch University

INSTRUCTIONS

(a) Explain the purpose of the study to the participants,

(b) Ask for consent before proceeding with the interview

(C) Make sure all questions are answered

(d) Circle choice of the answer as appropriate.

| **Part I** | **Socio demographic characteristics of the study participants** | |  |
| --- | --- | --- | --- |
| S.N | **Questions** | **Response** | Skip to…. |
| 1.1 | Respondent (household head)’s sex | 1. Female 2. Male |  |
| 1.2 | Age of household head in years. | ……………………………………………… |  |
| 1.3 | Marital status | .1. Single 2. Married  3. Divorced 4. Widowed 5.separated |  |
| 1.4 | Educational Status of the house hold head | 1. No formal education 2.Primary   3. Secondary 4. More than secondary |  |
| 1.5 | Occupation status of the household head. | 1. Farmer 2. House wife  3. Merchant 4. Daily laborer  5. Private employer  6. Other (specify) ___________ |  |
| 1.6 | Family size | 1. < 5 2. >5 |  |
| 1.7 | Are there Children whose age are (<18 years)? | 1. Yes 2. No |  |
| 1.8 | Are there elderly people in the household (65+ years)? | 1. Yes 2. No |  |
| **Part II** | **Physical accessibility of health facilities** | | |
| 2.1 | How long will it take to reach the nearest health center/hospital | 1. < 1 hour 2. 1-2 hour  3. > 2 hour |  |
| **Part III** | **Medical related factors** | | |
| 3.1 | Is there a person with chronic non communicable disease (hypertension, DM, heart disease, arthritis etc.) in the household? | 1. Yes 2. No |  |
| 3.2 | Is there frequently ill individual due to communicable disease in the household? | 1. Yes 2. No |  |

**Part IV: Community based health insurance knowledge related questions**

| S.N | Questions | Response | Skip to |
| --- | --- | --- | --- |
| 4.1 | Have you ever heard about community based health insurance program (CBHI)? [non-CBHI members only]  ? | Yes 2. No | ***If No s to q 4.3*** |
| 4.2 | If the answer for q4.1 is yes what is the main source of information? | 1= neighbors/friends  2= CBHI officials in public meeting  3= CBHI house to house awareness creation campaigns  4= mass media: TV, radio  5= health professionals in health facilities  6=others, specify____________________ |  |

[put ticks on the space provided]

| S.N | **Variables** | **Correct (1)** | **Not correct (2)** | **Do not know (3)** |
| --- | --- | --- | --- | --- |
| 4.3 | Only the very poor who cannot afford to pay for healthcare need to join the schemes. |  |  |  |
| 4.4 | Under CBHI program, you pay money (premiums) in order for the CBHI to finance your future health care needs? |  |  |  |
| 4.5 | CBHI program are like savings scheme, you will receive interest and get your money back. |  |  |  |
| 4.6 | If you do not make claims through CBHI, your premium will be returned. |  |  |  |
| 4.7 | Only those who fall sick should consider enrolment in CBHI. |  |  |  |

**Part V: Community based health insurance enrollment status**

| S.N | Questions | Response | Skip to. |
| --- | --- | --- | --- |
| 5.1 | Do You or other family members participated on any community based health insurance related meeting/training? | 1. Yes 2. No |  |
| 5.2 | Is your HH member enrolled in the CBHI program? | 1. Yes 2. No | If yes to Q5.6 |
| 5.3 | Why has your household decided not to enroll in the CBHI program (multiple responses allowed-list in order of importance)? | 1.Illness and injury does not occur frequently in our HH  2.the registration fee and premiums are not affordable  3 .want to wait in order to confirm the benefits of the scheme from others  4 .we do not know enough about the CBHI scheme  5 .there is limited availability of health services  6. the quality of health care services is low  7 . the benefit package does not meet our needs  8. Lack of confidence in scheme management  9.paying money for health care before illness is attracting diseases  10.other reasons, please specify |  |
| 5.4 | Do you plan to enroll in the CBHI scheme in the future? | 1.Yes 2. No |  |
| 5.5 | Is your household enrolled in any other solidarity group (e.g. iddir, equb, microfinance, other informal systems etc) active in your area? | 1.Yes 2. No |  |
| 5.6 | Are all members of your household enrolled in the CBHI program? | 1. Yes 2. No |  |
| 5.7 | If no to question 3.6, why are some members of the households are not enrolled in CBHI? | 1. members are healthy 2. do not have enough money to pay for all 3. members are not nuclear family members 4. others, specify |  |
| 5.8 | Why has your household or any of your household members decided to enroll in the CBHI program (multiple answers allowed-list in order of importance)? (CBHI enrolled only) | 1. Illness and/or injury occurs frequently in our HH 2. our HH members need health care 3. To finance health care expenses 4. CBHI registration and premium is paid by the government ***\|*** 5. Premium is low compared to the user fee price to obtain medical treatment ***\|*** 6. Pressure from other family members/community 7. Pressure from the kebele/tabia administration 8. others please specify |  |
| 5.9 | Who paid for the enrollment fee? | 1. household contribution  2. local government (coverage for Indigent households) |  |
| 5.10 | Where do you pay the premiums? | 1. At the CBHI office 2. Kebele/Tabia administration   3. Official comes and collects 4. others, please specify-------- |  |
| 5.11 | When you become CBHI member | ___________ |  |
| 5.12 | Which type of member you are? | 1. Indigent Member  2. Non-indigent Member |  |

**Part VI: Affordability and expectations**

| S.N | Questions | Response | Skip ... |
| --- | --- | --- | --- |
| 6.1 | The timing/time interval of premium payment is convenient for my household. | 1. Agree 2. Disagree |  |
| 6.2 | The CBHI registration fee is | 1.easily affordable  2. unaffordable |  |
| 6.3 | The CBHI regular contribution (premium) is | 1.easily affordable  2. unaffordable |  |
| 6.4 | Am very happy with current premium level? | 1. Agree   2. Disagree |  |

**Part VII: Community based health insurance (CBHI) experience**

(The following questions should be asked only to households who **are enrolled in the CBHI** program)

| S.N | Questions | Response | Skip .. |
| --- | --- | --- | --- |
| 7.1 | The local CBHI agent tries hard to solve CBHI implementation problems | 1. Disagree 2. indifferent 3. Agree |  |
| 7.2 | The community (CBHI members) has the right to guide and supervise the activities of the CBHI management | 1. Disagree 2. indifferent 3. Agree |  |
| 7.3 | The local CBHI management is trustworthy. | 1. Disagree 2. indifferent 3. Agree |  |
| 7.4 | I am satisfied with the experience at the local CBHI office when I go to register? | 1. Disagree 2. indifferent 3. Agree |  |
| 7.5 | I am satisfied with the local CBHI office when I go to pay the regular contribution (premium)? | 1. Disagree 2. indifferent 3. Agree |  |

# **Part VIII: House hold Economic Status**

| S,N | Question | Response | Remark |
| --- | --- | --- | --- |
| 8.1. | Source of drinking water? | 1. tap water 2. well  3. River water  4. other________ |  |
| 8.2. | Source of water other than drinking (washing dish, cooking…) | 1. Tap water 2. Well 3. River water   4. other________ |  |
| 8.3. | What type of latrine are you using? | 1. Pit latrine  2. Open field  3. other__________ |  |
| 8.4. | What is the source of energy for cooking food? | 1. Wood  2. charcoal  3. Electricity  4. other__________ |  |
| 8.5. | Do you have separate kitchen for cooking? | 1. yes 2. No |  |
| 8.6. | By what material the house floor is mainly made of? | 1. Earthen 2. cement  3. other__________ |  |
| 8.7. | By what material the wall is mainly made of? | 1. Mud / muck 2. stone  3. Clay 4. wood  5. Grass 6. other__________ |  |
| 8.8. | By what material the house roof is mainly made of? | 1. Corrugated iron 2. grass  3. other__________ |  |
| 8.9. | Do you have electricity in your house? | 1. Yes 2. No |  |
| 8.10. | Do you have radio in your house? | Yes 2. No |  |
| 8.11. | Do you have television in your house? | Yes 2. No |  |
| 8.12. | Do you have mobile phone in your house? | Yes 2. No |  |
| 8.13. | Do you have motor bicycle in your house? | Yes 2. No |  |
| 8.14. | How many bedrooms do you have in your house? | ------------- |  |
| 8.15 | Do you have agricultural land | Yes 2. No |  |
| 8.16. | If yes for ques 6. 15 ፣ how many hectare do you have? | ……………………….. |  |
| 8.17. | In how many hectares do have cultivated in the past 12 months?  (ask the crop items) | …………………. |  |
| 8.18. | Do you have domestic animal | Yes 2. No |  |
| 8.19. | If yes for ques 17 ፣ how many do you have? | cow ----------  equine -----------  Goat -----------  sheep -------------  hen -------------  beehive ---------- |  |
| 8.20. | Do you or your household member have a bank account or micro-finance book? | Yes 2. No |  |

**In-depth Interview guide**

Good morning/after noon, my name is______________________. I’m data collector of a research conducting on community based health insurance by Arba Minch University research team. The purpose of this assessment is to explore barriers of community based health insurance enrollment. This assessment will also provide recommendations for the scale-up of the pilot schemes to other districts. Any information you will provide as part of this interview will be held strictly confidential. Any reference to the information you provide in our analysis will be made without mentioning or implicating your name in any way. Our discussion will be audio recorded.

Participant’s signature______________________

Age_________________ Position____

1. What are the major successes and challenges in the scheme parameters?

a. Benefit package?

b. Level of premiums?

c. Membership scenario (household vs. individual base)

d. CBHI management schemes? Is there any complaint raised by members regarding these parameters?

2. The performance of schemes in terms of enrolment varies from region to region and from district to district? What do you think are the major factors for this regional and district variation in enrolments? Do you think CBHI will continue or not, do you think of other alternative?

3. Are there instances of fraud and abuse in CBHI financial resources management? If yes, what actions have been taken?

4. What are the challenges during community sensitization, what are the successes? What do you think about managerial commitment?

**FDG guiding tool**

**CBHI Members**

**INSTRUCTION TO THE INTERVIEWER**

Good morning/after noon, my name is______________________. I’m data collector of a research conducting on community based health insurance by Arba Minch University research team. The purpose of this assessment is to explore barriers of CBHI enrollment .This assessment will also provide recommendations for the scale-up of the pilot schemes to other districts. Any information you will provide as part of this interview will be held strictly confidential. Any reference to the information you provide in our analysis will be made without mentioning or implicating your name in any way. Our discussion will be audio recorded.

Participants’ signature_______________________________________

District: __________ FGD No: __________

Composition of FGD Participants:

1. Number of males:______ 2. Number of Females:__________ Date: _____________

1. What is community based health insurance? What is its benefit?

2. What types of households joined CBHI schemes and why?

3. Where did you get information regarding CBHI, from whom?

4. Indigent targeting - Some members of the CBHI get their contributions by the government as they are recognized as indigents.

a. Were you involved in the identification of indigents? Do you think the selection process is transparent and fair? Are there some people who are not included and others inappropriately included in the targeted groups? What do you suggest to improve the process?

5. What is the community’s perception regarding community based health insurance?

6. How the contributed money is collected, who collect the premium? Do you believe that the collected premium will be spent for your medical expenditure? Do you have trust on the management?

7. What do you think should be done to enroll the non-members of your community into the CBHI in your community? What should be adjusted to keep current members including you as a member of the scheme?

**NON-CBHI Members**

Good morning/after noon, my name is______________________. I’m data collector of a research conducting on community based health insurance by Arba Minch University research team. The purpose of this assessment is to explore barriers of CBHI enrollment .This assessment will also provide recommendations for the scale-up of the pilot schemes to other districts. Any information you will provide as part of this interview will be held strictly confidential. Any reference to the information you provide in our analysis will be made without mentioning or implicating your name in any way. Our discussion will be audio recorded.

1. Participants’ signature_______________________________________
2. District: __________ FGD No: __________

Composition of FGD Participants:

1. Number of males:______ 2. Number of Females:__________ Date: _____________

1. What is community based health insurance? What is its benefit?

2. Why did you decided not to join the CBHI scheme? Probe for culture &other reason including payment before illness

3. Do you know how much is paid by the CBHI members? What do you think about the affordability of the pre-payment scheme (registration fee and membership Fee)?

4. During the selection process of indigent members what mechanisms were used to ensure transparent and fair selection? Are there some people who are not included and others inappropriately included in the targeted groups? Do you think that all indigent people are included in the scheme?

5. How the contributed money is collected, who collect the premium? Do you have trust on the management?

6. What should be changed in the current CBHI scheme set up (payment levels, payment scheduling, benefit package, service availability etc.) to make you a member of a CBHI scheme

**ለቃለ መጠይቅ የፍቃደኝነት ውል**

እንደምን አደሩ/ዋሉ ስሜ --------------------ይባላል:: በአርባ ምንጭ ዩኒቨርስቲ የምርምር ቡድን በሚሰራ ማህበረሰብ አቀፍ የጤና መድን ፕሮግራም የተመለከተ ጥናት መረጃ ሰብሳቢ ነኝ፡፡ የጥናቱ አላማ ማህበረሰብ አቀፍ የጤና መድን ፕሮግራም ያለበት ደረጃ/ሁኔታ ለማወቅና ለመታቀፍ የሚያበረታቱ ወይንም ሊያግዱ የሚችሉ ሁኔታዎች ለማጥናት ሲሆን የርሶ ተሳትፎና ምላሽ ፕሮግራሙ የተሻለ ለማድረግ ጉልህ ሚና ይኖረዋል፡፡ እርሰዎ በአጋጣሚ ተመርጠዋል፡፡ መጠይቁ 20 ደቂቃ ይፈጃል፡፡ የሚሰጡት ምላሽ ሁሉ ሚስጢራዊ ሲሆን ከአማካሪዎቹ እና ከቃለ መጠይቅ አድራጊው ውጪ ጥቅም ላይ አይውልም፡፡ በዚህ ጥናት የሚሳተፉት በፈቃደኝነት ብቻ ነው፡፡ በቃለ መጠየቁ ግዜ ካልተመቾት በየትኛውም ሰአት ማቋረጥ ይችላሉ፡፡ በዚህ ጥናት በፈቃደኝነት ለመሳተፍ መስማማትዎን በቃል እንዲያረጋግጡልኝ በአክብሮት እጠይቃለሁ፡፡

ለመሳተፍ ፍቃደኛ ኖት? አዎ አይደለሁም

የተሳታፊው ፊርማ -----------------

የመረጃ ሰብሳቢው ስምና ፊርማ------------------------------------------------------------

የዋና ተመራማሪው አድራሻ፡ +251913976776, [mustesami02@gmail.com](mailto:mustesami02@gmail.com)

ምርምሩ ስፖንሰር ያደረገው ድርጅት ፡ አርባ ምንጭ ዩኒቨርስቲ

መመሪያ

(ሀ) የጥናቱ አላማ በደንብ አስረዳ ( ለ) ቃለ መጠይቅ ከማድረጎት በፊት ፈቃደኝነታቸው ጠይቅ

(መ) ሁሉንም ጥያቄዎች መመለሳቸው አረጋግጥ/ጭ (ሠ) በተገቢው መልኩ መልሱን አክብብ/ቢ.

| **ክፍል 1** | **፡ማህበራዊና ዲሞግራፊያዊ ሁኔታ በተመለከተ** | |  |
| --- | --- | --- | --- |
| ተ.ቁ | ጥያቄ | ምላሽ | ወደ ሚቀጥለው.. |
| 1.1 | ቤተሰብ ሀላፊ ፆታ | 1. ሴት 2. ወንድ |  |
| 1.2 | የቤተሰብ ሀላፊ ዕድሜ በዓመት | ……………………………………………… |  |
| 1.3 | የቤተሰብ ሀላፊ የጋብቻ ሁኔታ | 1. ያላገባ/ች 2. ያገባ/ች  3.የተፋታ/ች 4.የሞተበት/ባት 5.የተላየ/ች |  |
| 1.4 | የቤተሰብ ሀላፊ የትምህርት ሁኔታ | 1. ያልተማረ/ች 2. አንደኛ ደረጃ  3. ሁለተኛ ደረጃ 4. ከሁለተኛ ደረጃ በላይ |  |
| 1.5 | የቤተሰብ ሀላፊ የስራ ሁኔታ | 1.አርሶ አደር 2.የቤት እመቤት 3.ነጋዴ 4. የቀን ሰራተኛ  6. የግል ሰራተኛ 6. ሌላ ካለ ይገለፅ……………… |  |
| 1.6 | የቤተሰብ መጠን | 1. < 5 2. >5 |  |
| 1.7 | እድሜቸው ከ 18 ዓመት በታች የሆኑ ልጆች አሉ? | 1.አዎ 2. የለም |  |
| 1.8 | እድሜው የገፋ ሰው ቤት ዉስጥ አለ? (65 + አመት በላይ) | 1.አዎ 2. የለም |  |
| **ክፍል 2** | የጤና ተቋማት ቅርበት በተመለከተ | |  |
| 2.1 | የርሶ ቤትና ቅርብ ካለው ጤና ተቁም ምን ያህል ይርቃል? | 1. < 1 ሰአት 2. 1-2 ሰአት  3. > 2 ሰአት |  |
| **ክፍል 3** | የቤተሰብ የጤና ሁኔታ በተመለከተ | |  |
| 3.1 | በቤት ዉስጥ ተላላፊ ያልሆኑ በሽታዎች(ደም ግፊት፣ስኳር፣የልብ በሽታ ወዘተ..) ያለበት ሰው አለ? | 1. አዎ 2. አይደለም |  |
| 3.2 | የተለያዩ ህመሞች ማለትም በተላላፊ በሽታዎች ብዙ ጊዜ የሚታመም ሰው አለ? | 1. አዎ 2. አይደለም |  |

**ክፍል አራት ፡ማህበረሰብ አቀፍ የጤና መድን (ማአጤመ) እውቀት በተመለከተ**

| ተ.ቁ | ጥያቄ | ምላሽ | (ወደ ሚቀጥለው.. |
| --- | --- | --- | --- |
| 4.1 | ስለ ማህበረሰብ አቀፍ የጤና መድን ፕሮግራም ሰምቶ ያውቃሉ ? | 1. አዎ 2. አይደለም | መልሱ አይደለም ከሆነ ወደ ጥያቄ 4.3 ይለፉ |
| 4.2 | መልስዎ አዎ ከሆነ ከየት ነው የሰሙት | 1.ከጎረቤት /ከጓደኛ  2. ከማጤመ አስተባባሪዎች በስብሰባ ወቅት  3.ቤት ለቤት በተደረገ የግንዛቤ መስጨበጫ ቅስቀሳ  4.ከመገናኛ ብዙኃን፡ ሬድዮ፣ተለቪዥን 5.ከጤና ባለሙያዎች በጤና ተቋም ዉስጥ  6. ሌላ ካለ ይጠቀስ ይጠቀስ----------------- |  |

**መልሱን ቲክ አድርግ/ጊ**

| ጥያቄ | **ትክክል (1)** | **ትክክል አይደለም(2)** | **አላውቅም (3)** |
| --- | --- | --- | --- |
| 4.3.መታቀፍ ያለባቸው በጣም ድሀ የሆኑና የህክምና ወጪ መሸፈን የማይችሉት ብ ቻ ነው |  |  |  |
| 4.4. ለማጤመ ፕሮግራም የምትከፍሉት ክፍያ ለወደፊት የህክምና ወጪ እንዲያግዛቹ ነው |  |  |  |
| 4.5. ማጤመ ፕሮግራም እንደ ሌሎች ቁጠባ ማእቀፎች ወለድ የምታገኙበትና ገንዘባችሁ ይመለሳል |  |  |  |
| 4.6. ለማጤመ ፕሮግራም ጥያቄ/claim ካላቀረባችሁ የከፈላችሁት ገንዘብ ይመለሳል |  |  |  |
| 4.7. በማጤመ ፕሮግራም መታቀፍ ያለባቸው የታመሙት ብቻ ናቸው |  |  |  |

**ክፍል አምስት፡ የማጤመ ፕሮግራም ሁኔታ በተመለከተ**

| ተ.ቁ | ጥያቄ | ምላሽ | ወደ ሚቀጥለው.. |
| --- | --- | --- | --- |
| 5.1 | እርሶም ሆኑ ሌላ የቤተሰብ አባል ከ ማአጤመ ጋር የተገናኘ ስብሰባ/ስልጠና ተካፍለዋል? | 1.አዎ 2. አይደለም |  |
| 5.2 | ቤተሰብዎ የማአጤመ ፕሮግራም አባል ናቸው? | 1.አዎ 2. አይደለም | መልሱ አዎ ከሆነ ወደ ጥያቄ 5.6 ይለፉ |
| 5.3 | ለምንድነው ቤተሰብዎ በማጤመ ፕሮግራም ያልታቀፉት ? ( በቅደም ተከተላቸው/በደረጃ ከአንድ በላይ መልስ ይቻላል ) | 1.ህመምም ሆነ ጉዳት በቤተሰባችን ዉስጥ ብዙ ጊዜ አይከሰትም  2. ለ ማአጤመ መመዝገቢያና በየጊዜው የሚከፈለው ክፍያ ለመክፈል አቅሙ የለንም  3. በማአጤመ ለመመዝገብ ሌሎች ተጠቃሚዎች መጠቀማቸው አረጋግጠን ነው  4.ስለ ማአጤመ በቂ ግንዛቤ ስለሌለን ነው  5. በጣም ውስን የጤና አገልግሎት ስላለ ነው  6. በጤና ተቋም የሚሰጠው አገልግሎት ጥራት ስለሌለው  7. ከፕሮግራሙ የምናገኘው ጥቅም የአገልግሎት ፍላጎታችንን ስለማያሟላ  8. ፕሮግራሙ በሚያስተባብሩት እምነት ስሌለለን ነው  9.ህመም ከመከሰቱ በፊት ለጤና አገልግሎት ብር መክፈል በሽታ እንዲከሰት ያደርጋል  10.ሌላ ምክንያት ካለ ይጠቀስ--------------- |  |
| 5.4 | ወደፊት በማአጤመ ለመታቀፍ አቅደዋል? | 1.አዎ 2. አይደለም |  |
| 5.5 | ቤተሰብዎ በተለያዩ ማህበር ይሳተፋሉ ለምሳሌ እቁብ፣እድር፣ማይክሮ ፋይናንስ ፣ሌሎች ማህበር ወዘተ … | 1.አዎ 2. አይደለም |  |
| 5.6 | ሁሉም የቤተሰብዎ አባል በ ማአጤመ ፕሮግራም ታቅፍዋል | 1. አዎ 2. አይደለም |  |
| 5.7 | ለጥያቄ 3.5 መልስዎ አይደለም ከሆነ ለምን ቀሪዎቹ በ ማአጤመ አልታቀፉም | 1. ጤነኛ ስለሆኑ ነው  2. ለሁሉም ለመክፈል በቂ ብር ስለሌለ  3.ቀሪዎቹ የቅርብ ቤተሰብ ስላልሆኑ ነው  4. ሌላ ምክንያት ካለ ይጠቀስ--------------- |  |
| 5.8 | ለምንድነው ቤተሰብዎ የ ማአጤመ ፕሮግራም አባል የሆኑት( በቅደም ተከተላቸው/በደረጃ ከአንድ በላይ መልስ ይቻላል )  **ለማአጤመ አባላት ብቻ የሚጠየቅ** | 1.ብዙ ጊዜ በቤተሰባችን ዉስጥ ህመምና ጉዳት ስለሚከሰት  2. የጤና ተቋም አገልግሎት ስለምንፈልግ  3. የህክምና ወጪያችንን እንዲሸፍንልን  4. የ ማአጤመ መመዝገቢያና የሚከፈለው ክፍያ መንግስት ስለሚሸፍንልን  5. ለህክምና ከኪሳችን አውጥተን ከምንከፍለው ክፍያ በጣም ስለሚቀንስ  6. ከሌሎች ቤተሰቦች/ማህበረሰብ ግፊት  7.ከቀበሌና ጣቢያ አስተዳደር ግፊት  8. ሌላ ምክንያት ካለ ይጠቀስ--------------- |  |
| 5.9 | ለማአጤመ የሚከፈለው ክፍያ ማን ነው የሚከፍለው | 1. በቤተሰብ መዋጮ  2. የአካባቢው አስተዳደር ( በጣም ድሀ ለሆኑት ከሚሸፈነው) |  |
| 5.10 | ለማአጤመ ክፍያ የሚከፈለው የት ነው | 1. ማአጤመ ማስተባበሪያ ቢሮ 2. ቀበሌ/ጣቢያ አስተዳደር ቢሮ  3. ሀላፊዎች ቤት እየመጡ ይሰበስባሉ  4. ሌላ ካለ ይጠቀስ---- |  |
| 5.11 | የማአጤመ አባል መሆን የጀመሩት መቼ ነው? | ……………….ወራት ነው |  |
| 5.12 | ምን አይነት አባል ኖት? | 1.ሚስኪን 2. ሚስኪን ያልሆኑ |  |

**ክፍል ስድስት ፡የመክፈል አቅም እና የሰጡት ግምት**

በየ ስምምነት ደረጃችሁ የሚከተሉት አረፍተ ነገሮች ምላሽ ስጡባቸው

| ተ.ቁ | ጥያቄ | ምላሽ | ወደ ሚቀጥለው.. |
| --- | --- | --- | --- |
| 6.1 | የማአጤመ ክፍያ የሚከፈልበት ቀነ ገደብ ለቤተሰቤ ምቹ ነው | 1. እስማማለሁ 2.አልስማማም |  |
| 6.2 | የማአጤመ መመዝገቢያ ክፍያ | 1. በቀላሉ መክፈል የሚቻል ነው 2. መክፈል ሚቻል አይደለም |  |
| 6.3 | ለማአጤመ በየጊዜው የሚከፈለው ክፍያ | 1. በቀላሉ መክፈል የሚቻል ነው 2. መክፈል ሚቻል አይደለም |  |

**ክፍል ሰባት ፡ ከ ማአጤመ ልምድ በተመለከተ**

የሚከተሉት ጥያቄዎች የሚጠየቁት የማአጤመ **አባል ለሆኑት ብቻ ነው**

| ተ.ቁ | ጥያቄ | ምላሽ | Skip (ወደ ሚቀጥለው.. |
| --- | --- | --- | --- |
| 7.1 | የማአጤመ የአካባቢው ወኪል ማአጤመ ተግባራዊ እንዳይሆን የሚያደርጉ ችግሮችን ለመፍታት ይጥራል | 1. እስማማለሁ 2. ግድየለኝም 3. አልስማማም |  |
| 7.2 | የማአጤመ አባላት በማአጤመ አስተዳደራዊ ጉዳዮች ላይ አቅጣጫ ያሳያሉም ይደግፋሉ | 1. እስማማለሁ 2. ግድየለኝም 3. አልስማማም |  |
| 7.3 | የአካባቢ የማአጤመ አስተባባሪ አመኔታ ያተረፈ ነው | 1. እስማማለሁ 2. ግድየለኝም 3. አልስማማም |  |
| 7.4 | ለማአጤመ ለመመዝገብ በሄድኩበት ጊዜ በ ማአጤመ ቢሮ በገጠመኝ ነገር ረክቼያለሁ | 1.እስማማለሁ 2. ግድየለኝም  3. አልስማማም |  |
| 7.5 | ለማአጤመ መደበኛ ክፍያ ለመክፈል በሄድኩበት ጊዜ በ ማአጤመ ቢሮ በገጠመኝ ነገር ረክቼያለሁ | 1.እስማማለሁ 2. ግድየለኝም 3.አልስማማም |  |

**ክፍል ስምንት ፡ የቤተሰብ የሃብት ደረጃ መለኪያ ጥያቄዎች**

| ተ.ቁ | ጥያቄ | ምላሽ | አስተያየት |
| --- | --- | --- | --- |
| 8.1. | የመጠጥ ውሃ ከየት ነው ሚጠቀሙት? | 1. ቧንቧ ውሃ 2. የጉድጓድ ውሃ   1. ወራጅ ውሃ 4. ሌላ ካለ ይጠቀስ__________ |  |
| 8.2. | ከመጠጥ ዉጪ/ለሌላ አገልግሎት ማለትም ለመታጠብና ምግብ ለማብሰል የሚጠቀሙት ውሃ ከየት ነው ሚጠቀሙት | 1. ቧንቧ ውሃ 2. የጉድጓድ ውሃ  3.ወራጅ ውሃ 4. ሌላ ካለ ይጠቀስ__________ |  |
| 8.3. | ብዙ ጊዜ ቤተሰብዎ የሚጠቀሙት ምን አይነት መጸዳጃ ቤት ነው? | 1. ጉድጓድ 2. ሜዳ ላይ  3. ሌላ ካለ ይጠቀስ__________ |  |
| 8.4. | ምግብ ለማብሰል በዋናነት የሚጠቀሙት የሃይል ምንጭ ምንድነው ነው? | 1.እንጨት 2. ከሰል  3. ኤሌክትሪስቲ  4. ሌላ ካለ ይጠቀስ__________ |  |
| 8.5. | ምግብ ለማብሰል የተለየ ኩሽና አለዎት | 1. አዎ 2. አይደለም |  |
| 8.6. | የቤትዎ ወለል በዋናነት ከምንድነው የተሰራው | 1. ከአፈር 2. ሲሚንቶ  3. ሌላ ካለ ይጠቀስ__________ |  |
| 8.7. | የቤትዎ ግድግዳ በዋናነት ከምንድነው የተሰራው? | 1. ከጭቃ / ከኩበት 2. ከ ድንጋይ  3.ሲምንቶ/ከሸክላ 4. ከእንጨት  5.ከሳር  6. ሌላ ካለ ይጠቀስ__________ |  |
| 8.8. | የቤትዎ ጣሪያ በዋናነት ከምንድነው የተሰራው? | 1.ከ ቆርቆሮ 2.ከ ሳር  3. ሌላ ካለ ይጠቀስ__________ |  |
| 8.9. | በቤትዎ ዉስጥ ኤሌክትሪስቲ/ማብራት አለ | 1. አለ 2. የለም |  |
| 8.10. | በቤትዎ ዉስጥ ሬድዬ አለ | 1. አለ 2. የለም |  |
| 8.11. | እቤታችሁ ቴሌቪዥን አለ | 1. አለ 2. የለም |  |
| 8.12. | እቤታችሁ የሞባይል ስልክ አለ | 1. አለ 2. የለም |  |
| 8.13. | እቤታችሁ ሞተር ሣይክል አለ | 1. አለ 2. የለም |  |
| 8.14. | እቤታችሁ ስንት የመኝታ ክፍል አለ | ------------- |  |
| 8.15 | የእርሻ መሬት አላችሁ | 1. አለ 2. የለም |  |
| 8.16. | ለጥያቄ ቁጥር 15 አለ ከሆነ፣ ስንት ሄክታር ይሆናል | ……………………….. |  |
| 8.17. | ባለፈው 12 ወር ውስጥ በምን ያህል ሄክታር መሬት እህል አመረታችሁ የእህል አይነት ጠይቁ | …………………. |  |
| 8.18. | የቤት እንስሳ አላችሁ | 1. አለ 2. የለም |  |
| 8.19. | ለጥያቄ ቁጥር 17 አለ ከሆነ፣ ስንት አላችሁ | ከብት ----------  አህያ፣በቅሎ፣ፈረስ -----------  ፍየል ----------- በግ -------------  ዶሮ -------------የንብ ቀፎ ---------- |  |
| 6.20. | ከቤተሰባችሁ አባላት ውስጥ የባንክ ወይም የማይክሮፋይናንስ ደብተር ያለው ሰው አለ | 1. አለ 2. የለም |  |

**ቃለ መጠይቅ**

እንደምን አደሩ/ዋሉ ስሜ --------------------ይባላል ::በአርባ ምንጭ ዩኒቨርስቲ የምርምር ቡድን በሚሰራ ማህበረሰብ አቀፍ የጤና መድን ፕሮግራም የተመለከተ ጥናት መረጃ ሰብሳቢ ነኝ፡፡ የጥናቱ አላማ ማህበረሰብ አቀፍ የጤና መድን ፕሮግራም ያለበት ደረጃ/ሁኔታ ለማወቅና ና ለመታቀፍ የሚያበረታቱ ወይንም ሊያግዱ የሚችሉ ሁኔታዎች ለማጥናት ነው፡፡ የዳሰሳ ጥናቱ ማህበረሰብ አቀፍ የጤና መደን ፕሮግራም ወደ ላቀ ደረጃ ለማድረስና በዞን ደረጃ እንዲዳረስ አስተያየት ለመስጠት ጭምር ነው፡፡የሚሰጡት ምላሽ ሁሉ ሚስጢራዊ ሲሆን ከአማካሪዎቹ እና ከቃለ መጠይቅ አድራጊው ውጪ ጥቅም ላይ አይውልም፡፡ ማንኛውም እርሶ የሰጡን መረጃ ትንተና በምናደርግበት ጊዜ በምንም አይነት ሁኔታ ስሞን አንገልፅም፡፡ ድምጻችን ይቀዳል

ፍቃደኛ በመሆንዎ እናመሰግናለን ::

ፊርማ-------------

እድሜ_________________ የስራ ድርሻ____

1. የማአጤመ ፓራሜትር ዋና ዋና ስኬቶችና ተግዳሮቶች ምንድናቸው?

ሀ. ጥቅል አገልግሎት በተመለከተ?

ለ. የክፍያ መጠን?

መ. የአባልነት ሁኔታ (በቤተሰብ /በግለሰብ ደረጃ)?

ሰ. ማአጤመ አገልግሎት አስተባባሪ በተመለከተ (ማን፣እንዴት፣የት)

ረ. ሚስኪኖችን/የድሀ ድሃ እንዴት ነው የምትለዩት

እነዚህ በተመለከተ አባላቶቹ የሚያነሱት ቅሬታ አለ ?

2. የ ማአጤመ አፈፃፀም ከመታቀፍ/አባል ከመሆን አኳያ ከክልል ክልል ከወረዳ ወረዳ ይለያያል? የመለያቱ ምክንያት ምንድነው ትላለህ/ሽ? ማአጤመ ይቀጥል የሚል እምነት አለህ/ ወይስ ሌላ አማራጭ ቢሆር ብለህ ታስባለህ?

3. ለማአጤመ ተብሎ በሚሰበሰበው ብር ላይ የመመዝበርና የማጭበርበር አዝማሚያዎች ይታያሉ?

ካሉ ምን አይነት ርምጃዎች ተወስደዋል?

4. ህብረተሰቡ የማአጤመ አባል ለማድረግ በሚቀሰቀስበት ጊዜ ምን አይነት ውጤታማ ተግባሮች ነበሩ ፣ተግዳሮቶቹስ?

ምን ያህል ቤተሰብ ነው አባል የሆነው ፤ በወረዳ ደረጃ?

**ከባለ ድርሻ አካላት የሚደረግ ውይይት መነሻ ጥያቄዎች**

**አባል ለሆኑት ብቻ**

እንደምን አደሩ/ዋሉ ስሜ --------------------ይባላል ::በአርባ ምንጭ ዩኒቨርስቲ የምርምር ቡድን በሚሰራ ማህበረሰብ አቀፍ የጤና መድን ፕሮግራም የተመለከተ ጥናት መረጃ ሰብሳቢ ነኝ፡፡ የጥናቱ አላማ ማህበረሰብ አቀፍ የጤና መድን ፕሮግራም ያለበት ደረጃ/ሁኔታ ለማወቅና ና ለመታቀፍ የሚያበረታቱ ወይንም ሊያግዱ የሚችሉ ሁኔታዎች ለማጥናት ነው፡፡ የዳሰሳ ጥናቱ ማህበረሰብ አቀፍ የጤና መደን ፕሮግራም ወደ ላቀ ደረጃ ለማድረስና በዞን ደረጃ እንዲዳረስ አስተያየት ለመስጠት ጭምር ነው፡፡የሚሰጡት ምላሽ ሁሉ ሚስጢራዊ ሲሆን ከአማካሪዎቹ እና ከቃለ መጠይቅ አድራጊው ውጪ ጥቅም ላይ አይውልም፡፡ ማንኛውም እርሶ የሰጡን መረጃ ትንተና በምናደርግበት ጊዜ በምንም አይነት ሁኔታ ስሞን አንገልፅም፡፡ ድምጻችን ይቀዳል

ፍቃደኛ በመሆንዎ እናመሰግናለን ::

ፊርማ-----------------

ወረዳ: __________ የተሳታፊ ቁጥር: __________

የተሳታፊዎች ይዘት 1. የወንድ ብዛት:---------

2. የሴት ብዛት:__________ ቀን: _____________

1. ማአጤመ ምንድነው? ጠቀሜታውስ?

2. ምን አይነት ቤተሰብ ነው የ ማአጤመ አባል የሚሆነው እና ለምን?

3. ስለማአጤመ አጠቃላይ መረጃ ምታገኙባቸው ምንጮች

- ምን ምንድናቸው፣
- ምን አይነት እና ከማን

4. ሚስኪን/የድሃ ድሃ ተኮር፤አንዳንድ የማአጤመ አባላት ሚስኪን መሆናቸው ተረጋግጦ የአባልነት ክፍያ በመንግስት ይሸፈንላቸዋል

U. በምልመላ ሂደት ተሳትፎ አድርጋችሁ ነበር ?ሚስኪኖችን/የድሃ ድሃ በሚመረጥበት ጊዜ ግልጽኝነትና ፍትሀዊነት ለማረጋገጥ ሂደቱ ምን ይመስላል (ሚስኪን/የድሃ ድሃ በ ማአጤመ ስለመካተታቸው /ስላለመካተታቸው ያለችሁ አስተያየት ምን ይመስላል ፤ መክፈል እየቻሉ እንደማይችሉ ተደርጎ በመንግስት ድጋፍ የሚያገኙ ስለመኖራቸውና ስላለመሮቸው ምን አስተያየት አላችሁ? ሚስኪን/የድሃ ድሃ የሆኑ ሰዎች የመለየት ሂደት አንዲሻሻል ምን መደረግ አለበት ትላላችሁ?

5. ማህበረሰቡ ስለ ማአጤመ ጠቀሜታዎች ያሉት አመለካከት ምን ይመስላል?

6. ገንዘብ እንዴት ነው ሚሰበሰበው፣ማን ነው ሚሰበስበው?

- የሚሰበሰበው ብር በትክክል ለህክምናችን ይውላል ብላችሁ ታስባላችው? ገንዘብ በሚሰበስቡት ላይ እምነት አላችሁ ?

7. የማአጤመ አባል ያልሆኑት አባል እንዲሆኑ ምን መደረግ ያለበት ይመስላችኃል? በአሁን ጊዜ የማአጤመ አባል የሆኑት እናንተንም ጨምሮ አባል ሆናችሁ እንድትቆዩ መስተካከል ያለባቸው ምን ምንድናቸው?

**አባል ያልሆኑት ብቻ**

እንደምን አደሩ/ዋሉ ስሜ --------------------ይባላል ::በአርባ ምንጭ ዩኒቨርስቲ የምርምር ቡድን በሚሰራ ማህበረሰብ አቀፍ የጤና መድን ፕሮግራም የተመለከተ ጥናት መረጃ ሰብሳቢ ነኝ፡፡ የጥናቱ አላማ ማህበረሰብ አቀፍ የጤና መድን ፕሮግራም ያለበት ደረጃ/ሁኔታ ለማወቅና ና ለመታቀፍ የሚያበረታቱ ወይንም ሊያግዱ የሚችሉ ሁኔታዎች ለማጥናት ነው፡፡ የዳሰሳ ጥናቱ ማህበረሰብ አቀፍ የጤና መደን ፕሮግራም ወደ ላቀ ደረጃ ለማድረስና በዞን ደረጃ እንዲዳረስ አስተያየት ለመስጠት ጭምር ነው፡፡የሚሰጡት ምላሽ ሁሉ ሚስጢራዊ ሲሆን ከአማካሪዎቹ እና ከቃለ መጠይቅ አድራጊው ውጪ ጥቅም ላይ አይውልም፡፡ ማንኛውም እርሶ የሰጡን መረጃ ትንተና በምናደርግበት ጊዜ በምንም አይነት ሁኔታ ስሞን አንገልፅም፡፡ድምጻችን ይቀዳል

ፍቃደኛ በመሆንዎ እናመሰግናለን ::

ፊርማ-----------------

ወረዳ: __________ የተሳታፊ ቁጥር: __________

የተሳታፊዎች ይዘት 1. የወንድ ብዛት:---------

2. የሴት ብዛት:__________ ቀን: _____________

1. ማአጤመ ምንድነው? ጠቀሜታውስ?

2.የማአጤመ አባል እንዳይሆኑ ለምንድነው የወሰኑት? ባህልንም ጨምሮ ( ለህክምና አገልግሎት ቀድሞ ክፍያ በመክፈል ያለዎት አስተያየት ምንድነው)

3. የማአጤመ የአባልነት ክፍያ ምን ያህል እንደሆነ ያውቃሉ? የማአጤመ የመመዝገቢያ ና የአባልነት ክፍያ አቅምን ያገናዘበ ይመስሎታል?

4. ሚስኪኖችን/የድሃ ድሃ በሚመረጥበት ጊዜ ግልጽኝነትና ፍትሀዊነት ለማረጋገጥ ሂደቱ ምን ይመስላል (ሚስኪን/የድሃ ድሃ በ ማአጤመ ስለመካተታቸው /ስላለመካተታቸው ያለችሁ አስተያየት ምን ይመስላል ፤ መክፈል እየቻሉ እንደማይችሉ ተደርጎ በመንግስት ድጋፍ የሚያገኙ ስለመኖራቸውና ስላለመሮቸው ምን አስተያየት አላችሁ?

5. ገንዘብ እንዴት ነው ሚሰበሰበው፣ማን ነው ሚሰበስበው?

- የሚሰበሰበው ብር በትክክል ለህክምናችን ይውላል ብላችሁ ታስባላችው?

6**.** እርሶ አባል እንዲሆኑ ለማድረግ አሁን ካለው የማአጤመ አሰራር ና ደረጃ ምን መሻሻል አለበት ይላሉ (የክፍያ መጠንና አወሳሰን ፣የክፍያ ጊዜ፣ጥቅል ጠቀሜታዎች፣የአገልግሎት መኖር ወዘተ…)**?**
