# Supplementary material for: Factors affecting enrollment status of households for community based health insurance in a resource-limited peripheral area in Southern Ethiopia. Mixed method
Source: PLoS One. 2021 Jan 25;16(1):e0245952. doi: 10.1371/journal.pone.0245952 (PMC7833211; doi:10.1371/journal.pone.0245952)
Supplement: Attachment — Submitted filename: PONE-D-19-31045_reviewed.pdf [file pone.0245952.s003.pdf]

PLOS ONE

Adverse selection and supply-side factors in the enrollment of community-based health insurance in peripheral zones of South Nation Nationalities People Region, Ethiopia: Mixed Methodology

--Manuscript Draft--

Manuscript Number:	PONE-D-19-31045
Article Type:	Research Article
Full Title:	Adverse selection and supply-side factors in the enrollment of community-based health insurance in peripheral zones of South Nation Nationalities People Region, Ethiopia: Mixed Methodology
Short Title:	Adverse selection and supply-side factors in the enrollment of community-based health insurance
Corresponding Author:	Mustefa Glagn Abdilwohab, MPH<br>Arba Minch University<br>Arba Minch, ETHIOPIA
Keywords:	community based health insurance; enrollment; adverse selection; supply side factors; households
Abstract:	Background: Community based health insurance is the newly emerging not for profit type of insurance aims to reach and cover the very large rural agricultural sector and informal sector in urban settings, and to ensure universal health coverage. There is paucity of evidence on the enrollment status in Ethiopia in general and southern region in particular. Therefore, this study aimed to assess Enrollment Status of Households for community-based health insurance and identifying factors affecting it in peripheral zones of Southern Ethiopia.<br>Methods: A community based cross-sectional study was conducted among systematically selected 820 Households from April 27 to June 12, 2018. A pretested structured questionnaire, in-depth interview guide and Focus Group Discussion guiding tool were used to obtain information. A binary logistic regression model was used to assess the association between independent and outcome variables. Qualitative data was analyzed manually using thematic analysis method<br>Results: Out of 820 households 273(33.3%; 95% CI: 29.9- 36.2) were enrolled in the CBHI scheme. Among the non-enrolled households 44.1 % were reported that their reason not to enroll in CBHI was poor quality of service provided by public facilities. The odds of enrolling in CBHI among respondents who had good knowledge was 13.97 times higher than those with poor knowledge (AOR=13.97, 95%CI: 8.64, 22.60). In addition, households with elder, under 18 years children, frequently ill individual, presence of chronic disease in the household and educated household head showed positive association with CBHI enrollment<br>Conclusion and Recommendation: Community based health insurance status was low and showed a possibility of adverse selection in the scheme enrollment. Many more efforts are needed to enhance enrollment in CBHI by wide-ranging awareness creation activities through locally available social marketing strategies and a need to design different strategies to mitigate adverse selection. The intervention should also be focus on Enhancing managerial commitment, equipping health facilities with basic amenities and providing quality service for the potential beneficiaries.
Order of Authors:	Mustefa Glagn Abdilwohab, MPH
	Zeleke Hailemariam Abebo
	Wanzahun Godana Boyinto
	Desalegn Ajema
	Manaye Yihune
	Hadiya Hassen
Additional Information:	
Question	Response

Powered by Editorial Manager® and ProduXion Manager® from Aries Systems Corporation

**Financial Disclosure**<br><br>Enter a financial disclosure statement that describes the sources of funding for the work included in this submission. Review the submission guidelines for detailed requirements. View published research articles from *PLOS ONE* for specific examples.<br><br>This statement is required for submission and **will appear in the published article** if the submission is accepted. Please make sure it is accurate.<br><br>**Unfunded studies**<br>Enter: *The author(s) received no specific funding for this work.*<br><br>**Funded studies**<br>Enter a statement with the following details:<br>• Initials of the authors who received each award<br>• Grant numbers awarded to each author<br>• The full name of each funder<br>• URL of each funder website<br>• Did the sponsors or funders play any role in the study design, data collection and analysis, decision to publish, or preparation of the manuscript?<br>• **NO** - Include this sentence at the end of your statement: *The funders had no role in study design, data collection and analysis, decision to publish, or preparation of the manuscript.*<br>• **YES** - Specify the role(s) played.<br><br>* typeset	Arba Minch University is funding this research work with a project grant code of GOV/AMU/TH.3.1/CMHS/HO/02/10. The website of the university is http://www.amu.edu.et/. The funders had no role in study design, data collection and analysis, decision to publish, or preparation of the manuscript.
**Competing Interests**<br><br>Use the instructions below to enter a competing interest statement for this submission. On behalf of all authors, disclose any competing interests that could be perceived to bias this work—acknowledging all financial support and any other relevant financial or non-financial competing interests.<br><br>This statement **will appear in the published article** if the submission is accepted. Please make sure it is	The Author declare that there is no conflict of interest.

accurate. View published research articles from *PLOS ONE* for specific examples.<br><br>**NO authors have competing interests**<br><br>Enter: *The authors have declared that no competing interests exist.*<br><br>**Authors with competing interests**<br><br>Enter competing interest details beginning with this statement:<br><br>*I have read the journal's policy and the authors of this manuscript have the following competing interests: [insert competing interests here]*<br><br>* typeset	
**Ethics Statement**<br><br>Enter an ethics statement for this submission. This statement is required if the study involved:<br><br>• Human participants<br>• Human specimens or tissue<br>• Vertebrate animals or cephalopods<br>• Vertebrate embryos or tissues<br>• Field research<br><br>Write "N/A" if the submission does not require an ethics statement.<br><br>General guidance is provided below. Consult the submission guidelines for detailed instructions. **Make sure that all information entered here is included in the Methods section of the manuscript.**	Ethical approval was obtained from Institutional Ethics Review Board (IRB) of Arba Minch<br>University. Official permission letter was obtained from both Segen area and South Omo zonal Health department and the data collection begun after permission and cooperation letter was written to all three respective districts and respective kebele (the smallest administrative unit in Ethiopia) where the study was carried out. Household head informed written consent was obtained and the respondents were assured of confidentiality.

**Format for specific study types**<br><br>**Human Subject Research (involving human participants and/or tissue)**<br>• Give the name of the institutional review board or ethics committee that approved the study<br>• Include the approval number and/or a statement indicating approval of this research<br>• Indicate the form of consent obtained (written/oral) or the reason that consent was not obtained (e.g. the data were analyzed anonymously)<br><br>**Animal Research (involving vertebrate animals, embryos or tissues)**<br>• Provide the name of the Institutional Animal Care and Use Committee (IACUC) or other relevant ethics board that reviewed the study protocol, and indicate whether they approved this research or granted a formal waiver of ethical approval<br>• Include an approval number if one was obtained<br>• If the study involved *non-human primates*, add *additional details* about animal welfare and steps taken to ameliorate suffering<br>• If anesthesia, euthanasia, or any kind of animal sacrifice is part of the study, include briefly which substances and/or methods were applied<br><br>**Field Research**<br><br>Include the following details if this study involves the collection of plant, animal, or other materials from a natural setting:<br>• Field permit number<br>• Name of the institution or relevant body that granted permission	
**Data Availability**<br><br>Authors are required to make all data underlying the findings described fully available, without restriction, and from the time of publication. PLOS allows rare exceptions to address legal and ethical concerns. See the PLOS Data Policy and FAQ for detailed information.	Yes - all data are fully available without restriction

Powered by Editorial Manager® and ProduXion Manager® from Aries Systems Corporation

A Data Availability Statement describing where the data can be found is required at submission. Your answers to this question constitute the Data Availability Statement and **will be published in the article**, if accepted.<br><br>**Important:** Stating 'data available on request from the author' is not sufficient. If your data are only available upon request, select 'No' for the first question and explain your exceptional situation in the text box.<br><br>Do the authors confirm that all data underlying the findings described in their manuscript are fully available without restriction?	
**Describe where the data may be found in full sentences. If you are copying our sample text, replace any instances of XXX with the appropriate details.**<br><br>• If the data are **held or will be held in a public repository**, include URLs, accession numbers or DOIs. If this information will only be available after acceptance, indicate this by ticking the box below. For example: *All XXX files are available from the XXX database (accession number(s) XXX, XXX.)*.<br>• If the data are all contained **within the manuscript and/or Supporting Information files**, enter the following: *All relevant data are within the manuscript and its Supporting Information files.*<br>• If neither of these applies but you are able to provide **details of access elsewhere**, with or without limitations, please do so. For example:<br><br>*Data cannot be shared publicly because of [XXX]. Data are available from the XXX Institutional Data Access / Ethics Committee (contact via XXX) for researchers who meet the criteria for access to confidential data.*<br><br>*The data underlying the results presented in the study are available from (include the name of the third party*	All relevant data are within the manuscript and its Supporting Information files.

and contact information or URL).<br>• This text is appropriate if the data are owned by a third party and authors do not have permission to share the data.<br><br>* typeset	
Additional data availability information:	

Powered by Editorial Manager® and ProduXion Manager® from Aries Systems Corporation

Manuscript Click here to download Manuscript CBHI manuscript.docx

Adverse selection and supply-side factors in the enrollment of community-based health insurance in peripheral zones of South Nation Nationalities People Region, Ethiopia: Mixed Methodology

Mustefa Glagn Abdilwohab[1*], Zeleke Hailemariam Abebo [2], Wanzahun Godana Boyinto [3], Dessalegn Ajema [4] Manaye Yihune [5] Hadiya Hassen[6]

1,2,4,5,6 Department of Public Health, College of Medicine and Health Sciences, Arbaminch University, Arbaminch, Ethiopia.

3 Department of Public Health Arba Minch University, Ethiopia and University of Ghent, Belgium

1* Corresponding Author: Mustefa Glagn Abdilwohab, Email: mustesami02@gmail.com, Phone: +251-913-976776

Abstract

Background: Community based health insurance is the newly emerging not for profit type of insurance aims to reach and cover the very large rural agricultural sector and informal sector in urban settings, and to ensure universal health coverage. There is paucity of evidence on the enrollment status in Ethiopia in general and southern region in particular. Therefore, this study aimed to assess Enrollment Status of Households for community-based health insurance and identifying factors affecting it in peripheral zones of Southern Ethiopia.

Methods: A community based cross-sectional study was conducted among systematically selected 820 Households from April 27 to June 12, 2018. A pretested structured questionnaire, in-depth interview guide and Focus Group Discussion guiding tool were used to obtain information. A binary logistic regression model was used to assess the association between independent and outcome variables. Qualitative data was analyzed manually using thematic analysis method

Results: Out of 820 households 273(33.3%; 95% CI: 29.9- 36.2) were enrolled in the CBHI scheme. Among the non-enrolled households 44.1 % were reported that their reason not to enroll in CBHI was poor quality of service provided by public facilities. The odds of enrolling in CBHI among respondents who had good knowledge was 13.97 times higher than those with poor knowledge (AOR=13.97, 95%CI: 8.64, 22.60). In addition, households with elder, under 18 years children, frequently ill individual, presence of chronic disease in the household and educated household head showed positive association with CBHI enrollment

Conclusion and Recommendation: Community based health insurance status was low and showed a possibility of adverse selection in the scheme enrollment. Many more efforts are needed to enhance enrollment in CBHI by wide-ranging awareness creation activities through locally available social marketing strategies and a need to design different strategies to mitigate adverse

selection. The intervention should also be focus on Enhancing managerial commitment, equipping health facilities with basic amenities and providing quality service for the potential beneficiaries.

Introduction

Primary health care financing as one of the structural aspects of health systems that play an essential role in ensuring universal health coverage (UHC) (1,2). The goal of UHC as backed by the World Health Organization (WHO) is to eliminate the financial difficulty associated with obtaining the necessary health services that ensure the wellbeing and productivity of a society. Mechanisms that offer health security through risk pooling like a Community Based Health Insurance (CBHI) Scheme is one of the possible tools in achieving UHC (1). CBHI) is part of the Ethiopian government broader health care financing reform strategy aims to promote equitable access to health care, increase financial protection, promote cost sharing between the government and citizens, and enhance domestic resource mobilization for the health sector and social inclusion in health (3,4). It is supposed to reduce unforeseeable or unaffordable healthcare costs (in the case of illnesses) to regularly paid premiums (5-7). Following this approach and after sharing experience from forefront countries in CBHI, in June 2011, with the aim of enhancing access to health care and reducing the burden of out-of-pocket health care expenditure, the Government of Ethiopia rolled out a pilot CBHI scheme. The scheme aimed to serve rural households and urban informal sector workers in 13 districts situated in four main regions (Tigray, Amhara, Oromiya, and South Nation Nationalities People Region) of the country. After three years of piloting, the government decided to expand CBHI schemes to 161 districts of the country (8). The overall enrollment rate of households for CBHI in the pilot districts of Ethiopia in 2013 was 52.4% (9). However, only about 1.2% of the Ethiopian citizens had health insurance from both private and public agencies (10). Despite having over a decade of experience in the sub Saharan region, it is not prevalent in Eastern African countries; Tanzania, Uganda and Kenya are countries in east Africa which have experiences with CBHI though their coverage has not reached more than 15%

(11), a high rate of coverage is seen in Rwanda where coverage has scaled up from 35% in 2006 to 85% in 2008 (12).

Meta-analysis suggests that enrollments in CBHI were positively associated with household income, education of the head of households, age of the head of the household, household size, female-headed household, trust in the scheme management, and married head of the household and presence of chronic illness episodes in the household (13). Generally, the existing health insurance coverage in Sub-Saharan region is very low and it is vulnerable to adverse selection (13,14). Even though, Ethiopia has been implementing CBHI scheme since 2011 to promote health of poor rural and urban informal residents, enrollment rate is still very low when compared to the potential beneficiaries. Majority of the research conducted in Ethiopia are focused on willingness to pay for CBHI. There is a single study on the CBHI enrollment at national level in the pilot district (8). Specifically, to our study area the socio-cultural context is very different from those in the pilot woreda and they were not included in the pilot study. In addition, the factors are not well described in Ethiopia in general and the study setting in particular. Therefore, this study aimed to assess Enrollment Status of Households for community-based health insurance and identifying factors affecting it. So, a better understanding of the sustainability, coverage, and associated factors could be helpful to make the case for policy and program interventions and to direct resources most effectively and efficiently.

Study design, setting and population

Community-based cross-sectional study with both quantitative and qualitative method of data collection was conducted during April 27 to June 12, 2018 among households living in community-based health insurance implemented districts of Segen area and south Omo zones, southern Ethiopia. Household head and/or spouse who were working in the formal sectors were

excluded. Both South Omo and Segen area zones are located in the peripheral zones of southern region. There were eight and four districts in south Omo and Segen area zones respectively. Among which four districts and one district were implementing community-based health insurance during the study period in South omo and Segen area zone respectively. The administrative offices of the zones are located about 750 KMs south of Addis Ababa. In the settings of this study, subsistence agriculture is the main economic activity, and the income of majority of the households depends on agriculture. But one of the district's population are semi-pastoralist and Pastoralist and their main income depends on livestock.

Sample size and sampling technique

Sample size was determine by using single population proportion formula, taking assumptions (95% confidence level, 5% margin of error) and by taking 52.4 % enrollment status of community based health insurance on pilot CBHI implementation Woredas in Ethiopia, and using design effect of two and adding 10% non-response rate, sample size for the number of households under study was 842 (9). Multi-stage sampling technique was employed to reach the study participants. There were four fully CBHI implemented woreda in South Omo zone, and one fully CBHI implemented woreda in Segen area zone. First, fifty percent (50%) of the districts with in South Omo zone in which, community-based health insurance implemented districts were included in the study by using simple random sampling technique; and one woreda that fully implemented CBHI was taken directly from Segen area zone. Secondly, 25% of kebeles with in the selected districts were included in the study by using simple random sampling technique. Finally, households were selected by using systematic sampling technique after proportionally allocated to the kebele based on the size.

Data collection tools and procedures

Data were collected using face to face interviewer administered structured, pretested Amharic version questionnaires. The respondents were the head of the households. The study Instruments/tools for this study was adapted from federal democratic republic of Ethiopian health insurance agency, a pretested evaluation tool (8). The data on household wealth index was collected by asking ownership of selected assets based on EDHS 2016 wealth index variables (15).

Qualitative data collection

Focus Group Discussion guiding tool and In-depth Interview guide were also used to collect qualitative data. The data collectors were Master of Public Health holders with experience of data collection and fluent speakers of local language. The Key informants: three heath office heads, three woreda CBHI coordinators and 9 Health Extension Workers were interviewed. The in-depth interview was conducted for 30-35 minutes, and the place of interview was at the office. Each interview was audio-recorded. Two FGD in each woreda, one for CBHI member and one for nonmember was conducted. A total of six FGDs (with a group of 7-11) were conducted. FGD was conducted for 90 minutes and audio recorded. There was also informal conversation, unstructured spontaneous discussions with the participants, to get the opportunity to ask pertinent questions on different occasions. This could minimize the possibility of participants altering their response purposefully or holding back information on sensitive issues like disclosing any attempt on abuse or fraud of the collected money but that are important to our study.

Data quality control

A pretest was done prior to actual data collection by recruiting 42 households out of the study setting but with similar socio-economic context. The final version of the questionnaire was translated into Amharic language and again translated back to English to check the consistency. The data collectors and supervisors were given two days of intensive training on the overall data collection procedure, ethical issues and the purpose of the study. After the pretest relevant modifications were made before the commencement of the actual data collection. Supervisors have checked the collected data for completeness and consistency throughout the data collection period.

Data analysis

Data were entered into Epi Data version 3.1. After checking and correcting errors, the data were exported to Statistical Package for Social Science (SPSS) version 21 software for further analysis. Descriptive analysis of data was indicated using numerical summary measures. Outliers were also checked. Household wealth index was computed using a composite indicator by considering properties like, livestock ownership, selected household assets and agricultural land. Principal component analysis (PCA) was performed to categorize the household wealth index. The level of analysis for this study was the household, considering that enrollment in Ethiopian CBHI is currently at the household level. The outcome variable was treated as a binary outcome (1 for enrolled and 0 for not enrolled households). Binary logistic regression was carried out to assess the association of different independent Variables with the dependent variable after assumptions of logistic regression were checked. Independent variables having $P \leq 0.25$ on simple binary logistic regression analysis were Considered as candidates for the multivariable logistic regression analysis. The final model was fitted using backward Conditional variable selection methods and Hosmer and Lemeshow Test of model adequacy was 0.90. Multivariable logistic regression

analysis was carried out to identify factors having statistically significant associations with the dependent variable. Finally, the adjusted odds ratio (AOR) with a 95% confidence interval was used to determine the association between CBHI enrollment status and the independent variables.

Qualitative data analysis: data were collected, transcribed, translated, coded and analyzed manually using thematic analysis method; and finally, qualitative data results were presented with quantitative result through triangulation by using narrative weaving approach. Quotes that were most useful in explaining the quantitative findings were selected and fond of under each quantitative finding.

Ethical considerations

Ethical approval was obtained from Institutional Ethics Review Board (IRB) of Arba Minch University. Official permission letter was obtained from both Segen area and South Omo zonal Health department and the data collection begun after permission and cooperation letter was written to all three respective districts and respective kebele (the smallest administrative unit in Ethiopia) where the study was carried out. Household head informed written consent was obtained and the respondents were assured of confidentiality.

Results

Socio- demographic and economic characteristics of the study participants

A total of 842 participants/house hold head were recruited, while 820 (97.38%) consented to participate in the present study. The mean age of the participants was 40.0 ± 11.05 years (SD) with age range between 20-76 years. Out of the study participants 43.7% had no formal education and 62.4% of them had a family size of less than or equal to five (see Table 1).

Table 1: Socio- demographic and economic characteristics of respondents (n=820) at Community Based Health Insurance Implemented Districts of Segen area and South Omo zones, April 27 to June 12, 2018.

Variables	Category	frequency	Percent (%)
Age of head of the household	<=25 years	58	7.1
	26-34 years	225	27.4
	35-44 years	250	30.5
	45-54 years	186	22.7
	Above 55 years	101	12.3
Sex of the participant (household head)	Female	147	17.9
	Male	673	82.1
Educational status Of head of the household	No formal education	358	43.7
	Primary school	344	42.0
	Secondary school	55	6.7
	Above Secondary	63	7.7
Marital status of head of the household	Single	41	5.0
	Married	665	81.1
	Divorced	39	4.8
	Widowed	58	7.1
	separated	17	2.1
Occupational status of head of the household	Farmer	468	57.1
	pastoralist	115	14.0
	Merchant	161	19.6
	Daily laborer	40	4.9
	other	36	4.4
Family size	=< 5	512	62.4
	>5	308	37.6
Wealth quantile	Lowest	133	16.2
	Second	104	12.7
	Middle	402	49.0
	Fourth	20	2.4
	Highest	161	19.6
Children age less than 18 years	yes	497	60.6
	No	323	39.4
	Yes	179	21.8

Elderly people in the household (65+ years)	No	641	78.2

Medical related factor

Out of the study participants 127(15.5%) reported that one or more of the household members had non communicable disease. Of them 84.25 % households were enrolled in community-based health insurance scheme. One hundred forty-six (17.8%) of the study participants reported that there was an episode of illness due to communicable disease among one or more members. Among the household who were encountered episode of communicable disease 82.8% of them were enrolled in community-based health insurance.

Physical accessibility of health facilities

When examining the time taken to reach the nearest health facilities, 37.3%, 59% and 3.7% of the participants reported that they were spending less than or equal to one hours, one to two hours and greater than or equal to two hours to reach the facilities and receiving health care respectively.

Knowledge of the study participants regarding CBHI

A total of 561/820 (68.4%) of the respondents reported having heard about community-based health insurance messages. Of those who had heard about CBHI messages 47.41% were enrolled in the scheme. Source of information see the detail in figure 1.

Table 2: Responses about the Role and Concept of CBHI (%)

variables	Response		
	**Correct**	**Not correct**	**Do not know**
Only the very poor who cannot afford to pay for healthcare need to join the schemes ***(Incorrect)***	128(15.6%)	400(48.8%)	292(35.6%)
Under CBHI program, you pay money (premiums) in order for the CBHI to finance your future health care needs ***(Correct)***	489(59.6%)	45(5.5%)	286(34.9%)
CBHI program are like savings scheme, you will receive interest and get your money back ***(Incorrect)***	88(10.7%)	412(50.2%)	320(39.0%)
If you do not make claims through CBHI, your premium will be returned ***(Incorrect)***	84(10.2%)	391(47.7%)	345(42.1%)
Only those who fall sick should consider enrolment in CBHI ***(Incorrect)***	83(10.1%)	440(53.7%)	297(36.2%)

Negatively worded variables were reworded before calculating the overall Knowledge score. A total of 374 (45.6 %) of the study participants had good knowledge. FGD response about concept and understanding of CBHI: *"Although we heard about MA'TEMMA local name of 'community-based health insurance' from our village, no body from government or any other concerned bodies, told us comprehensive importance, benefit packages and even its meaning." (FDG-Non CBHI members)*

Community Based Health Insurance (CBHI) Enrollment Status

Out of the study participants 273(33.3%; 95% CI: 29.9- 36.2) were enrolled in the CBHI scheme, while the remaining 547(66.7%) were not enrolled. A total of 289 (35.2%) of the respondent reported that they or the member of the family were participated on any community-based health insurance related meeting/training whereas 531(64.8%) were not participated. Reasons forwarded by the household decided not to enroll in the CBHI program: see the detail in figure 2.

Reason not to enroll in CBHI forwarded by FDG participants *"The care given to us at the hospital/health center is poor, when we go to hospital or health center, we haven't been getting medication even for headache. …health professionals write prescription to the private drug pharmacies….in this situation, how can we be CBHI member? Even we haven't getting quality service by our pocket payment. We don't think, we can get adequate service for free…. let's see whether those previously enrolled members get the service or not…. after that we will be a member." (FDG-Non CBHI members).*

Among the respondents 279 (34.0%) of them were enrolled in social solidarity group (e.g. idir, equb, microfinance, other informal systems etc.) active in their area.

Table 2:Reasons forwarded by enrolled households for Enrolling in the CBHI Implemented Districts of Segen area and South omo zones, April 27 to June 12, 2018 (n=273).

Reason	frequency	%
Illness and/or injury occurs frequently in our HH	151	55.3
Our HH members need health care	146	53.5
To finance health care expenses	232	85.0
Premium is low compared to the user fee price to obtain medical treatment |	173	63.4
Pressure from other family members/community	23	8.4
Pressure from the kebele/tabia administration	98	35.9

Affordability of the premium and expectations from CBHI program

Only 279 (34.0%) of the respondents agree that the time of collection of the regular premium is convenient for their households. But 93.2% and 93% of the participants reported that both registration fee and regular premium are easily affordable respectively.

One of the female FGD participant explained by manifesting her pleasant face, "the premium we have contributed is easily affordable---if you go to private clinic, with in a single visit you can pay 1000 birr or more. …when you compare CBHI with the private clinic payment it is nil. …it is a

gift from the government to the poor (who cannot afford to pay) for medical expenditure…. the problem is the service is not started yet.”

Table 3: Community Based Health Insurance experience of the enrolled households in the CBHI Implemented Districts of Segen area and South omo zones, April 27 to June 12, 2018 (n=273).

**variables**	**Response**		
	Agree	Indifferent	Disagree
The local CBHI agent tries hard to solve CBHI implementation problem	164(60.1%)	3(1.1%)	106(38.8%)
The community (CBHI members) has the right to guide and supervise the activities of the CBHI management	89(32.6%)	42 (15.4%)	142(52.0%)
The local CBHI management is trustworthy	104 (38.1%)	58 (21.2%)	111 (40.7%)
I am satisfied with the experience at the local CBHI office when I go to register?	36 (13.2%)	13 (4.8%)	224 (82.1%)
I am satisfied with the local CBHI office when I go to pay the regular contribution (premium)	58 (21.2%)	25 (9.2%)	190 (69.6%)

The response forwarded by the FDG participants about trust in Scheme management: *“Because of lower level management system had previous history of fraud and corruption on public resources, currently we have no trust on them. …now the government is sending them to join higher education without any punitive measures….in addition we did not see the fruit of previously contributed money for different purposes…genuinely speaking we are not thinking of not to be fraud or abused our contributed premium for CBHI.” (FDG- CBHI member)*

Community involvement

We (the investigators) have observed there were no strong involvement of the community, i.e., community leaders, religious leaders, elders, and others in addition to government structures at

woreda and kebele levels in sensitization and awareness-creation activities that can facilitate the acceptance of the schemes and increased enrollment rates.

"We have no idea how much birr is collected; how many households were enrolled…where the premium is pooled…. simply they had taken our money but the service is not started yet…we need our money to pay back otherwise the service should be started." (FGD-CBHI member)

Linkage of community-based health insurance enrollment and associated factors

Educational status, sex, age, marital and occupational status of the household head, family size, Presence of children whose age are (≤18 years) & elders (65+ years), presence of a person with chronic disease (NCDs) & frequently ill individual due to communicable disease, Knowledge and distance from the health facility were eligible for multivariable logistic regression.

In multivariable binary logistic regression Knowledge, family size, Presence of children whose age are (≤18 years) & elders (65+ years), presence of a person with chronic disease (NCDs) & frequently ill individual due to communicable disease and educational status of the household head remained to have an association with CBHI enrollment. However, distance from the health facility sex, age and occupational status of the household head did not show statistically significant association with CHBI enrollment. The odds of enrolling in CBHI among families who had a family member of greater than five was 1.88 times higher than family member of five or less (AOR=1.88,95% CI: 1.15, 3.06)

The odds of enrolling in CBHI among households who had children whose age are (≤18 years) was 3.64 times higher than families with no children under 18 years (AOR=3.64, 95% CI: 2.09, 6.33). The odds of enrolling in CBHI among households with the presence of elders was 2.60 times higher than without elders (AOR=2.60, 95%CI: 1.45, 4.65). The odds of enrolling in CBHI among respondents who had good knowledge was 13.97 times higher than those with poor knowledge

(AOR=13.97, 95%CI: 8.64, 22.60). The odds of enrolling in CBHI among households with a presence of a person with chronic disease in the household was 3.64 time higher than households with no chronic disease (AOR=3.64, 95% CI: 1.67, 7.79). The odds of enrolling in CBHI among household head who had primary education 3.06 times higher than with no formal education (AOR=3.06, 95 % CI: 1.88, 4.99). The odds of enrolling in CBHI among frequently ill individual due to communicable disease in the household was 3.90 times higher than with no frequently ill person in the household (AOR= 3.90, 95% CI:2.03, 7.51).

Table 4: Multivariable logistic regression to identify the associated factor of community-based health insurance enrollment among households living in the CBHI Implemented Districts of Segen area and South omo zones, April 27 to June 12, 2018.

**Variables**		Enrolled In CBHI	Not enrolled in CBHI	COR (95% CI)	AOR (95% CI)
Family size	<= 5	86 (16.8%)	426(83.2%)	1.00	1.00
	>5	187(60.7%)	121(39.3%)	7.65(5.52,10.60)	1.88(1.15, 3.06)
Children whose age are (≤18 years) are present in the HH	yes	243(48.9)	254(51.1%)	9.34(6.17,14.14)	3.64(2.09, 6.33)
	No	30(9.3%)	293(90.7%)	1.00	1.00
elderly people in the household (65+ years) are present in the HH	yes	128(71.5%)	51(28.5%)	8.58(5.90,12.47)	2.60(1.45, 4.65)
	No	145(22.6%)	496(77.4%)	1.00	1.00
a person with chronic disease in the household	yes	107(84.3%)	20(15.7%)	16.98(10.21,28.23)	3.64(1.67, 7.79)
	No	166(24.0%)	527(76.0%)	1.00	1.00
frequently ill individual due to communicable disease in the household	Yes	121(82.9%)	25(17.1%)	16.62(10.42,26.51)	3.90(2.03, 7.51)
	No	152(22.6%)	522(77.4%0	1.00	1.00
Knowledge	good	240(64.2%)	134(35.8)	22.41(14.83,33.86)	13.97(8.64,22.60)
	poor	33(7.4%)	413(92.6%)	1.00	1.00
Educational status of the household head	No formal education	87(24.3%)	271(75.7%)	1.00	1.00
	primary	153(44.5%)	191(55.5%)	2.45(1.81,3.44)	3.06(1.88, 4.99)
	secondary	16(29.1%)	39(70.9%)	1.27(0.07,2.39)	.98(.37, 2.59)
	Above secondary	17(27.0%)	46(73.0%)	1.15(0.62,2.1)	1.12(.49, 2.66)

Note: HH=household; AOR=Adjusted odd Ratio; COR= Crude odd ratio, p-value< 0.05

Supply side factors from qualitative findings

Lack of managerial commitment

"There is a great gap in managerial commitment …the manager think that it is the only role of the health extension workers…I have been trying my best to make the community to be the member of the scheme. ...Some of our community are scatter and pastoralist as well as there are hard to reach kebeles. … we need strong managerial support….the community named me 255 (two hundred fifty five) "hulet hamsa amist" because of complaining that... you took our money but the service is not started yet." (HEWs focal).

Inconsistent CBHI implementation and premium level

Most of the health extension workers complaining that there is no consistent premium level and CBHI implementation strategies in different woreda even in the kebeles (smallest administrative unit)

Fraud and abuse of the premium

"The health extension workers and the kebele administrators (who are responsible for collecting the premium in the study area) they have been using the collected money for their own sake and some of them lend the money to others and some of them make a business using it." (In-depth -interview: Woreda CBHI-coordinator).

Policy issues related to CBHI

"One of the main bottle neck not to start CBHI service is government regulation …Because of 50 % of the woreda eligible households are not member, we have not started providing the service for previously enrolled households….For me, the solution is that either to start in any number of households or making membership obligatory."(In-depth -interview: WoHO head).

Discussion

Enrollment rate of the households in Community Based Health Insurance was (33.3%; 95% CI: 29.9- 36.2) in the present study. This finding was lower when compared with the study conducted in the Rwanda 85% (12), pilot woreda of Ethiopia (52.4%) (8), Northwest part of Ethiopia in one of the pilot woreda 42% (16), and Tanzania 49% (17). This difference might be attributed to the lower involvement of influential community members: community leaders, religious leaders, elders and health development army in sensitization and awareness-creation activities. The other probable reason for lower involvement might be lack of managerial commitment at the lower level. We are suggesting the engagement of the community in decision-making about the types of services, Payment approach and service delivery so that it would have a great contribution for increasing enrollment in the scheme. The other reason for low reason might be due to political instability in the study areas. The result of the present study is used as a signal for a need of much efforts to attain the goal of Ethiopian government health sector transformation plan to expand CBHI schemes to 80% of woredas and enroll at least 80% of households by 2020 (18).

The current study showed that Knowledge regarding community-based health insurance positively affect the decision of enrollment. The odds of enrolling in CBHI among respondents who had good knowledge was 13.97 times higher than those with poor knowledge. This result is analogous to study findings in the other corner of Ethiopia (19) and other African countries like Uganda, Kenya, and Nigeria; the evidences in those countries showed limited information and poor knowledge limit voluntary enrollment and re-enrollment in the scheme. (11, 20, and 21). The concept of insurance and risk pooling is relatively new for many people in low-income countries in general and remote area like our study setting in particular. We can increase CBHI understanding and concept using different social marketing strategies including locally available means; and efficient

information campaign as well as provision of training on the important parameters of CBHI, would contribute on the improvement of understanding and knowledge of the community on health insurance and definitely will increase the enrollment rate. Moreover, the odds of enrolling in CBHI among household head who had primary education was 3.06 times higher than with no formal education. This is also corroborated by the study finding about willingness to join CBHI in Ethiopia (22), enrollment studies in Kenya, India and Bangladesh (20, 23, 24). The finding can be explained as those educated household heads' are likely to have better acceptance and knowledge about the meaning as well as the benefit package of CBHI and its protection from catastrophic out of pocket health expenditure at the time of ailment, which leads them to make a rational decision to enroll in the scheme. While, the study done in Tanzania show that there is no significant relation between education and CBHI enrollment (17).

In this study, households with larger size had higher odds of CBHI enrollment than those with a smaller number of family members. The finding was in agreement with the study conducted in Ethiopia (16). This can be justified as there was no variation with premium payment among different family size in Ethiopia except in Oromiya region, it is advantageous for households with larger family size to join the scheme. Household unit of enrollment is can be effective mechanism to address all family members. In addition, household enrollment decreased adverse selection due to lower probability of having only sick and higher risk individuals enrolled in the scheme. In contrast, the three studies show that households with larger size are less likely to enroll in CBHI the reason might be due to there was member restriction on the mentioned studies and they might face difficulties in meeting the subscription fees (11, 25,26). The present study also illustrates that household members with chronic disease, frequently ill individual in the household and house hold with under eighteen children and elders are more likely to enroll in the scheme than their counter

parts. Even though, the scheme aimed to facilitate health service utilization and promote equitable distribution of health service among different segment of the population. Such practice may endanger risk sharing principles, as those who have medical conditions and elders who have a probability of getting sick frequently involve actively, thus increase chance of using all the resource within a short period of time exhaustively; and the result also show us the existence of adverse selection problem. This evidence also supported by the research done in Thehuldere, north east part of Ethiopia (27) and the two-study conducted in India (23,28).

In Nigeria, Uganda and Rwanda (6, 11,29) (members and nonmembers of CBHI schemes complained about the inconvenient facility like lack of drugs and supplies affect their enrollment. This finding also corroborated by the qualitative finding of the present study, the FGD participants Nonmember of CBHI complained that *"there is no medication, even medication to treat headache in public facilities"*. Moreover, the other study conducted in Low- and Middle-Income Countries including Nigeria (6, 30) the result showed the perception of the community towards good quality healthcare provided in the public facilities or availability of quality service as a factor enhancing enrolment. This is also supported by the current study, 44.1 % of the participant reported that their main reason for not to enroll in CBHI scheme was poor quality of service provided in the public health facilities. This was also verified by FGD nonmember participants, "*The care given to us at the hospital/health center is poor, and how we can be a member?"* This could reflect negative attitude towards public facilities, meanwhile the community have good opinion towards CBHI, if that is the case, the government body would be better to fulfill the facilities and the recommended services should be provided for the community, so as to more member will be enrolled in the CBHI scheme.

The finding of study conducted in Uganda and Tanzania (11, 17), financial support from government and managerial commitment were reported to have a positive influence on CBHI enrollment and sustaining the scheme and sufficiently meeting the health needs of the communities. So, this result highlight lack of managerial commitment affects the CBHI enrollment. This evidence is supported by the qualitative finding of the present study, one of the CBHI coordinator complain that the main barrier not to enroll the community in the scheme was "*lack of managerial commitment at different level*". He also suggests the involvement of academician on the development process of CBHI scheme. Moreover, our finding also shows that there was lower involvement of the community on decision making regarding CBHI, fifty two percent of the participant reported that the community has no the right to guide and supervise the activities of the CBHI management. This result was also supported by FGD participant," *We have no idea how much birr is collected, how many households were enrolled…where the premium is pooled..."* This finding reflects, the community-based ideology from which the CBHI was built is missing according to the responses from community members. Clearly, it indicates lack of solidarity and risk sharing principles.

Conclusion and Recommendation

The study found that lower enrollment rate of the community in community Based Health Insurance scheme. The evidence from this study showed that certain factors affecting voluntary uptake of CBHI are basically demand-driven. Knowledge, family size, Presence of children whose age are (≤18 years) & elders (65+ years), presence of a person with chronic disease (NCDs) & frequently ill individual due to communicable disease and educational status of the household head were positively influence CBHI enrollment. This result showed that there was adverse selection in the study area, these should be properly addressed in scheme development process and

implementation and keeping pace across the different levels to ensure attainment of scheme objectives. The study also identified lower managerial commitment, lower involvement of the community in the decision making regarding CBHI scheme management. Moreover, insufficient information among nonmember and mismanagement of the collected money was also reported in this study. The other evidence generated from the study was the perception that healthcare is of poor quality in public health facilities and lack of facilities including drugs and supplies deter the community to enroll in CBHI. In general, low enrollment in the CBHI scheme in the study area does not necessarily indicate low demand by potential enrollees. The enrollment rate can be increased if the service is started with the available number of already enrolled members. Many more efforts are needed to enhance enrollment in CBHI by wide-ranging awareness creation activities through locally available social marketing strategies and considering different enabling and deterring socio-demographic factors. The intervention should also be focus on Enhancing managerial commitment, equipping health facilities with basic amenities and providing quality service for the potential beneficiaries.

Funding: Arba Minch University is funding this research work with a project grant code of **GOV/AMU/TH.3.1/CMHS/HO/02/10**. The website of the university is http://www.amu.edu.et/. The funders had no role in study design, data collection and analysis, decision to publish, or preparation of the manuscript.

Acknowledgments

First, we would like thank all study participants, data collectors, supervisors and study area Zonal Health Departments & District Health Offices. Second, we are very thankful for Arba Minch University, College of Medicine and Health Science staffs for their constructive advice and support. We are also very thankful to Arba Minch University for funding our study.

Author Contributions:

Conceptualization: Mustefa Glagn Abdilwohab and Zeleke Hailemariam Abebo

Data curation: Mustefa Glagn Abdilwohab

Formal analysis: Mustefa Glagn Abdilwohab

Methodology: Mustefa Glagn Abdilwohab , Zeleke Hailemariam Abebo , Wanzahun Godana Boyinto , Dessalegn Ajema, Manaye Yihune ,Hadiya Hassen

Funding acquisition: Mustefa Glagn Abdilwohab

Writing ± original draft: Mustefa Glagn Abdilwohab and Zeleke Hailemariam Abebo

Writing ± review & editing: Mustefa Glagn Abdilwohab, Zeleke Hailemariam Abebo, Wanzahun Godana Boyinto , Dessalegn Ajema, Manaye Yihune and Hadiya Hassen

Competing Interest: The author declares that there is no competing interest

References

1. World Health Organisation (WHO). Together on the road to universal health coverage 2017;[Internet].Available from: http://www.who.int/universal_health_coverage/road-to-uhc/en/%0Ahttp://apps.who.int/iris/bitstream/10665/258962/1/WHO-HIS-HGF-17.1 eng.pdf?ua=1
2. WHO. The world health report: health systems financing: the path to universal coverage, Geneva. 2010:15-50).
3. Solomon F, Hailu Z, Tesfaye D. A. Ethiopia's Community-based Health Insurance: A Step on the Road to Universal Health Coverage. Heal Financ Gov [Internet]. 2011;12. Available from: https://www.hfgproject.org/ethiopias-community-based-health-insurance-step-road-universal-health-coverage/
4. Chankova, Slavea, Sara Sulzbach, Francois Diop "Impact of mutual health organizations: evidence from West Africa. "Health Policy and Planning. 2008; 23(4):268-75.
5. Tabor SR. Community-Based Health Insurance and Social Protection Policy. March 2005:13-
6. Onwujekwe, S Eze, N Ezuma, EN Obikeze and CA Onoka: Implementing Community Based Health Insurance in Anambra State, Nigeria. 2010.
7. World Bank .Health Financing Revisited, A Practitioner's Guide. The International: 2014.
8. Ethiopian Health Insurance Agency. Evaluation of Community-Based Health Insurance Pilot Schemes in Ethiopia: Final Report. 2015;(May):21. Available from: http://www.jointlearningnetwork.org/resources/download/get_file/ZW50cnlfaWQ6MzgyOHxmaWVsZF9uYW1lOnJlc291cmNlX2ZpbGV8dHlwZTpmaWxl%0Ahttps://www.hfgproject.org/evaluation-cbhi-pilots-ethiopia-final-report/
9. Feleke S, Mitiku W, Zelelew H, et al. Ethiopia's Community-based Health Insurance: A Step on the Road to Universal Health Coverage, 2015.

10. Wang W, Temsah G. Mallick L: Health insurance coverage and its impact on maternal health care utilization in low- and middle-income countries, in DHS Analytical Studies No. 45, Vol. *Rockville, Maryland,USA, ICF International* 2014.

11. Basaza R, Criel B, Van der Stuyft P. Community health insurance in Uganda: why does enrolment remain low? A view from beneath. *Health Policy Plan*. 2008; 87(2):172-184.
12. Shemeles A. community-based health insurance scheme in Africa, the case of Rwanda, working paper. Africa development bank. 2012 (120):13-7.
13. Pradeep Panda et al. Factor affecting uptake of voluntary and community-based health insurance scheme in middle and low-income countries: A systematic review, 2016.
14. Wang W, Temsah G. Mallick L: Health insurance coverage and its impact on maternal health care utilization in low- and middle-income countries, in DHS Analytical Studies No. 45, Vol. *Rockville, Maryland,USA, ICF International* 2014.
15. ICF. CSA (CSA) [Ethiopia] and. Central Statistical Agency (CSA) [Ethiopia] and ICF. Ethiopia Demographic and Health Survey 2016. Addis Ababa, Ethiopia, and Rockville, Maryland, USA: CSA and ICF. 2016.
16. Atafu A, Kwon S. Adverse selection and supply-side factors in the enrollment incommunity-based health insurance in Northwest Ethiopia: A mixed methodology. *Int J Health Plann Mgmt*.2018;1–13. https://doi.org/10.1002/hpm.2546
17. Kapologwe et al. Barriers and facilitators to enrollment and re-enrollment into the community health funds/Tiba Kwa Kadi (CHF/TIKA) in Tanzania: a cross-sectional inquiry on the effects of socio-demographic factors and social marketing strategies: BMC Health Services Research (2017) 17:308
18. Ethiopian Federal Ministry of Health. Health Sector Transformation Plan (2015/16-2019/20). 2015;20(May):1–118.
19. Mebratie AD, Sparrow R, Yilma Z, Alemu G, Bedi AS. Dropping out of Ethiopia's community-based health insurance scheme. *Health Policy Plan*. 2015;30(10):1296–1306.
20. . Kiplagat I, Muriithi M, Kioko U. Determinants of health insurance choice in Kenya. *Eur Sci J*. 2013;9(13).

21. Banwat M, et al. Community based health insurance knowledge and willingness to pay; a survey of a rural community in north central zone of Nigeria. *Jos Journal of Medicine*. 2012;6(1):54-59.
22. Haile M, Ololo S, Megersa B. Willingness to join community-based health insurance among rural households of Debub Bench District, Bench Maji Zone, Southwest Ethiopia. *BMC Public Health*. 2014;14(1):591.
23. . Panda P, Chakraborty A, Dror DM, Bedi AS. Enrolment in community-based health insurance schemes in rural Bihar and Uttar Pradesh, India. *Health Policy Plan*. 2014;29(8):960-974.
24. Ahmed S, Hoque ME, Sarker AR, et al. Willingness-to-pay for community-based health insurance among informal workers in Urban Bangladesh. *PLoS one*. 2016;11(2):e0148211.
25. Gnawali DP, Pokhrel S, Sie A, Sanon M, De Allegri M, Souares A, Dong H,Sauerborn R. The effect of community-based health insurance on the utilization of modern health care services: evidence from Burkina Faso.Health Policy. 2009; 90:214–22.
26. Mladovsky P, Soors W, Ndiaye P, Ndiaye A, Criel B. Can social capital help explain enrolment (or lack thereof) in community-based health insurance?Results of an exploratory mixed methods study from Senegal. Soc Sci Med.2014; 101:18–27.
27. Workneh SG, Biks GA and Woreta SA:Community-based health insurance and communities' scheme requirement compliance in Thehuldere district, northeast Ethiopia: Dove press Journal: ClinicoEconomics and Outcomes Research, 2018.
28. Aggarwal A. Achieving equity in health through community-based health insurance: India's experience with a large CBHI Programme. Kobe: Discussion Paper Series-DP2010-31, RIEB, Kobe University;2010 Nov 10.
29. Schneider P. Trust in micro-health insurance: an exploratory study in Rwanda. Soc Sci Med. 2005; 61:1430–8.
30. Dror DM, Hossain SAS, Majumdar A, Pérez Koehlmoos TL, John D, Panda PK What
31. Factors Affect Voluntary Uptake of Community-Based Health Insurance Schemes in Low- and Middle-Income Countries? A Systematic Review and Meta-Analysis. PLoS ONE, (2016) 11(8): e0160479. doi:10.1371/journal.pone.0160479

figure Click here to download Figure Figures.docx

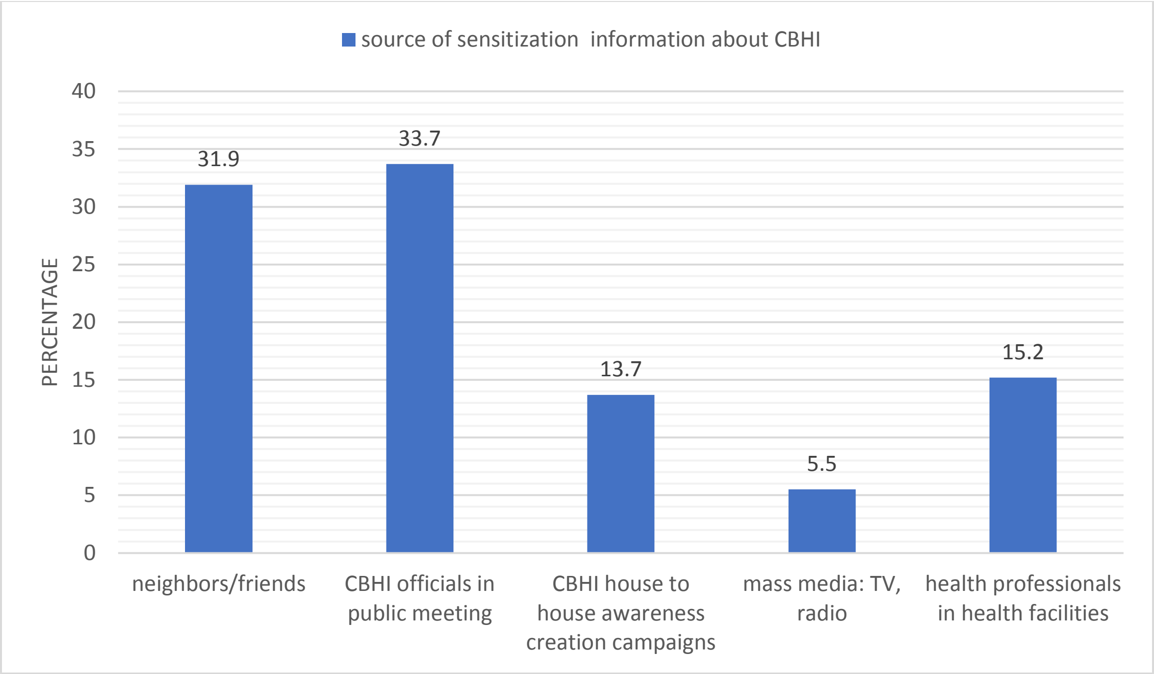

Figure 1: Shows source of sensitization information regarding Community Based Health Insurance Implemented Districts of Segen area and South omo zones, April 27 to June 12, 2018.

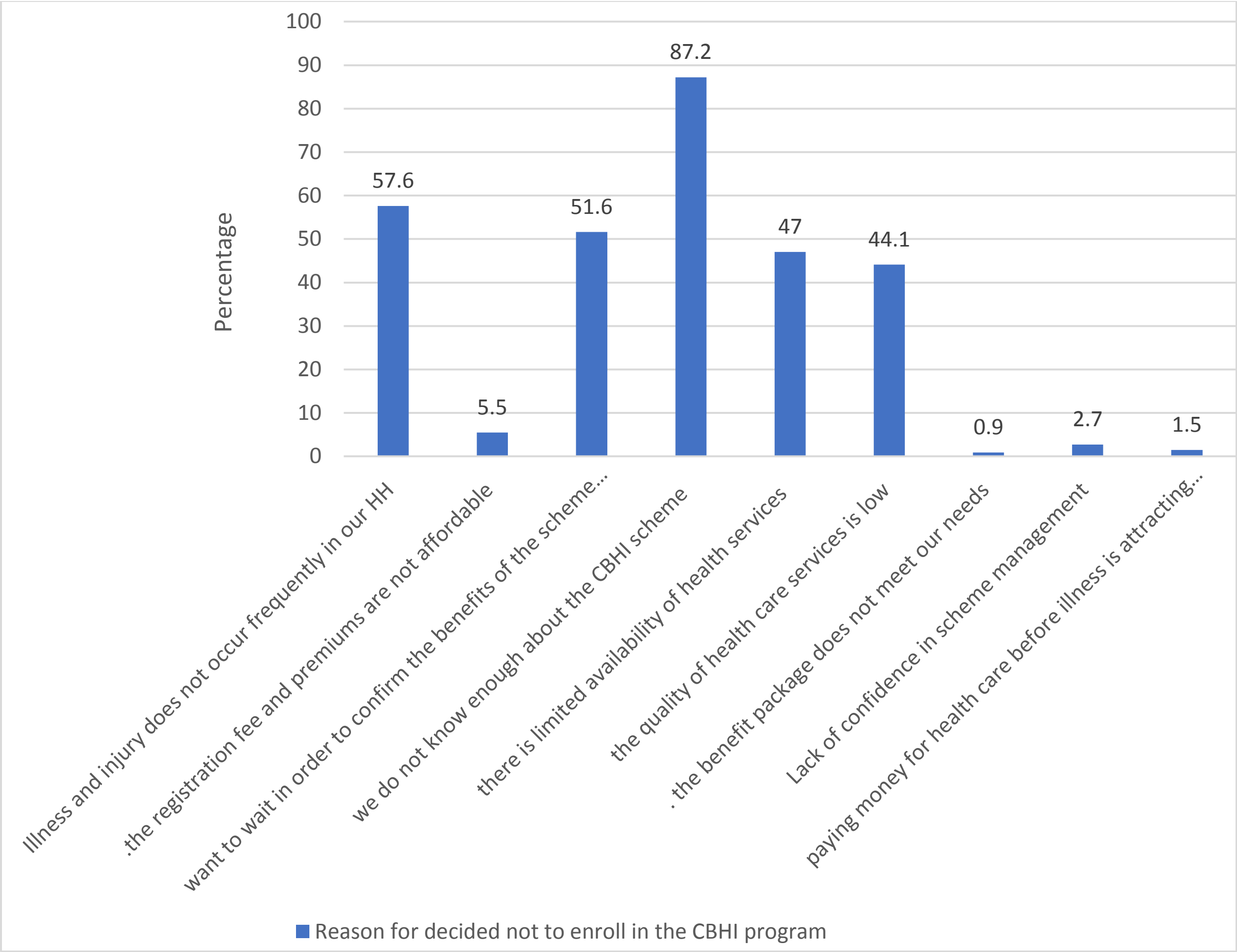

Figure 2: Reason for decided not to enroll in the Community Based Health Insurance Implemented Districts of Segen area and South omo zones, April 27 to June 12, 2018 (n=547).

raw data

Click here to access/download
Supporting Information
Mustefa cbhi.spss -.sav
