# Supplementary material for: Factors affecting enrollment status of households for community based health insurance in a resource-limited peripheral area in Southern Ethiopia. Mixed method
Source: PLoS One. 2021 Jan 25;16(1):e0245952. doi: 10.1371/journal.pone.0245952 (PMC7833211; doi:10.1371/journal.pone.0245952)
Supplement: Attachment — Submitted filename: Author response to reviewers.docx [file pone.0245952.s004.docx]

**Authors response for Editor**

Marta Pascual
Academic Editor
PLOS ONE

Journal Requirements:

When submitting your revision, we need you to address these additional requirements.

1. Please ensure that your manuscript meets PLOS ONE's style requirements, including those for file naming. The PLOS ONE style templates can be found at

http://www.journals.plos.org/plosone/s/file?id=wjVg/PLOSOne_formatting_sample_main_body.pdf and <http://www.journals.plos.org/plosone/s/file?id=ba62/PLOSOne_formatting_sample_title_authors_affiliations.pdf>

**Response:** We have revised the manuscript using PLOS ONE guideline and checked the contents included in the guideline.

2. Please address the following:

- Please modify the title to ensure that it is meeting PLOS’ guidelines (https://journals.plos.org/plosone/s/submission-guidelines#loc-title). In particular, the title should be "specific, descriptive, concise, and comprehensible to readers outside the field".  Please ensure that you amend the title both on the online submission form (via Edit Submission) and the title in the manuscript so that they are identical.

**Response:** **Title** **editing effected accordingly**

Factors affecting enrollment status of households for community based health insurance in a resource-limited peripheral area in Southern Ethiopia.

We have amended the title both on online submission form (via Edit Submission) and the title in the manuscript.

- Please ensure you have thoroughly discussed any potential limitations of this study within the Discussion section.

Response: Kindly, included the limitations of the study

- Please include additional information regarding the survey or questionnaire used in the study and ensure that you have provided sufficient details that others could replicate the analyses. For instance, if you developed a questionnaire as part of this study and it is not under a copyright more restrictive than CC-BY, please include a copy, in both the original language and English, as Supporting Information.

Response: kindly, we have attached the questionnaire used in the study as Supporting Information.

Thank you for your attention to these queries.

3. Please include a separate caption for each figure in your manuscript.

Response:  Kindly, included a separate caption for each figure in the manuscript

4. We note you have included tables to which you do not refer in the text of your manuscript. Please ensure that you refer to Tables 2, 3 and 4 in your text; if accepted, production will need this reference to link the reader to each Table.

Response: We appreciate for your meticulous observation of the error we made in reporting.

We have revised the whole manuscript and corrections were made accordingly.

We have referred all the tables in our text.

5. Please include captions for your Supporting Information files at the end of your manuscript, and update any in-text citations to match accordingly. Please see our Supporting Information guidelines for more information: <http://journals.plos.org/plosone/s/supporting-information>

Kindly, included the captions for Supporting Information files at the end of the manuscript.

[Note:HTMLmarkup is below. Please do not edit.]

Reviewers'comments:

Reviewer's Responses to Questions

**Authors response for reviewers questions**

**For reviewer 1:**

**Response**: Title editing effected accordingly

Factors affecting enrollment status of households for community based health insurance in a resource-limited peripheral area in Southern Ethiopia.

**Q1. The study presents the results of original research.**

The concern of reviewer is on similarity with Atafu et al., published article. But there are significant differences between our study and the study mentioned above.

1. Atafu et al used the tool to measure the variables from undefined source but we adapted enrollment status and the factors from the tool used in Ethiopian health insurance agency. <https://www.hfgproject.org/evaluation-cbhi-pilots-ethiopia-final-report/>
2. Our study is done in resource limited peripheral districts in Southern Ethiopia, which also helps to generate evidences from minorities. Moreover, this areas are usually neglected by researchers for different studies because of their remote nature to physical accessibility which also affects health research inequity to address information need to evidence based practice

**Q2. Results reported have not been published elsewhere.**

**Response:** Here the concern of reviewer is duplication of paper with different studies. In fact, the study from North West Ethiopia is somehow similar with the study we conducted but three things should be taken in to consideration.

1. As mentioned above the study setting is quite different. In Ethiopian context, there are accessible and inaccessible areas for health services and information. Accordingly our research addresses the equity issue related to adequate information to intervene public health problem which is related to alternative financing mechanism.
2. Our study has exhaustively addressed supply side factors by qualitative method which usually is not addressed by many studies including the mentioned study in Ethiopia
3. Other studies mentioned are out of Ethiopia and even their variable of interest is on willingness to enroll, not on enrollment status

**Q3. Experiments, statistics, and other analyses are performed to a high technical standard and are described in sufficient detail**.

**Response**: We really appreciated the reviewers concern regarding lack of synthesis for the quotes. Kindly, the reviewer can find the themes, synthesis and quotes for each synthesis in qualitative data presentation. Kindly, see the details under ‘qualitative data analysis’ section of the manuscript.

Otherwise the qualitative data were analyzed by thematic analysis method. Therefore no quantitative categorization and calculating χ^2^ are applicable for qualitative data.

***The authors referred to a principal comment analysis (PCA), but the results of such analysis are not shown anyway in the manuscript. Also, when using relatively unfamiliar data analysis methods such as PCA for basic analysis, the authors should give a rationale**.

**Response:** This study was conducted in rural setting in which households cannot clearly define their wealth status. Therefore, in our study principal component analysis was used to calculate the composite wealth index by considering different assets. Kindly refer the result of PCA under Table 1 …wealth quantile which was categorized as lowest, second, middle, fourth and highest.

**The authors correctly focus on logistic regression analysis for most part of the data analysis. However, they still some confusion. The authors state that all variables from univariable regression with a p-value less or equal to 2 were put back into a multivariable regression, but there are no p-values shown anywhere in the tables or in the body of this manuscript.**

**Response:***Kindly the correction is effected

Here is the final multivariable logistic regression model

| **Variables in the Equation** | | B | S.E. | Wald | df | Sig. | Exp(B) | 95% C.I.for EXP(B) | |
| --- | --- | --- | --- | --- | --- | --- | --- | --- | --- |
|  |  |  |  |  |  |  |  | Lower | Upper |
| Step 6^a^ | FAMS(1) | .630 | .250 | 6.364 | 1 | .012 | 1.877 | 1.151 | 3.062 |
|  | CHILD(1) | 1.293 | .282 | 20.990 | 1 | .000 | 3.643 | 2.095 | 6.332 |
|  | ELDER(1) | .956 | .296 | 10.421 | 1 | .001 | 2.602 | 1.456 | 4.651 |
|  | NCOMBLED(1) | 1.291 | .389 | 11.024 | 1 | .001 | 3.638 | 1.697 | 7.796 |
|  | ILCOMBLE(1) | 1.362 | .334 | 16.612 | 1 | .000 | 3.902 | 2.027 | 7.510 |
|  | klgecat(1) | 2.637 | .245 | 115.469 | 1 | .000 | 13.975 | 8.639 | 22.608 |
|  | EDUS |  |  | 22.872 | 3 | .000 |  |  |  |
|  | EDUS(1) | 1.120 | .249 | 20.264 | 1 | .000 | 3.065 | 1.882 | 4.992 |
|  | EDUS(2) | -.012 | .493 | .001 | 1 | .981 | .988 | .376 | 2.595 |
|  | EDUS(3) | .139 | .430 | .104 | 1 | .747 | 1.149 | .495 | 2.666 |
|  | Constant | -4.542 | .342 | 176.735 | 1 | .000 | .011 |  |  |

a. Variable(s) entered on step 1: SEX, MARITALS, OCCN, FAMS, CHILD, ELDER, DISTANCE, NCOMBLED, ILCOMBLE, agecat, klgecat, EDUS.

**On page 12, the authors described about 12 variables that were returned into the multivariable analysis model, but "Table 4" (which supposed to be Table 5), only show 7 variables.**

**Response:** Thank you the reviewer for your meticulous observation for improving our paper.

Table numbers mislabeled were kindly corrected. These seven variables were the variables which were included in the final model using backward regression. For this reason we have presented only the variables which remained in the final model.

**The tables are miss-numbered (for example we have two 'table 2s', and so on), contain a tremendous amount of structural, grammatical and spelling errors that distract the reader from following. These tables could benefit as traditionally done; making the description of the variables shorter in the table, and then use of a table legend to describe the abbreviations. Table 1 needs some structural and grammatical editing.**

**Response**: Kindly correction is effected

**The data in Table 2 is contradictory form the author's claim in the text. Row# 3 and 4 in table 2 demonstrate that if you combine the percentages of participants who answered incorrectly and those who did not know, that numbers is higher than those who answer correctly, suggesting that in general participants did not understand the concept or did not know how the CBHI system work. However, in the paragraph below the table, the authors only use the percentage from the “correct” column to conclude that participants have a good knowledge and understanding of CBHI.**

**Response:** The result we presented is correct but we found that it is confusing for the reader. The results which will be presented in the manuscript should be easily understandable by the readers. So that now we made it simple and clear.

*Thank you for your observation, we corrected it on the table as ‘correctly responded’ and ‘incorrectly responded’ for those who responded right answer and wrong answer respectively.

For the mathematical calculation of knowledge of the participants is well explained under operational definition in method section. Kindly, you can get the answer for your question related to knowledge.

**4. Conclusions are presented in an appropriate fashion and are supported by the data.**

**The conclusions are supported by the data but are obvious and similar or exactly the same to most recent publications that the authors either are not way of or failed to acknowledge. It will beneficial to the field if the authors relate their results to these most recent publications.
• The authors failed to make an important point that keeps appearing from the testimonials of participants. It seems like the CBHI administrators have failed to manage the expectation of the community. In some testimonials, participants are admitting that the premium is very affordable but the public hospitals/clinics services are very bad. The simple solution could be to educate the community that they can only be able to get the services that the CBHI is able to pay for regarding the low premium. It will be beneficial to the recommendations if the authors speculate on that point.**

**Response:** *Correction is effected as follows:

Conclusion section in the Manuscript: There was lower enrollment status of the households in community based health insurance (CBHI) scheme. We found that enrollment to community based health insurance was higher among people who had good knowledge, higher family size, presence of children whose age (≤18 years) and or elders (65+ years), presence of a household member with chronic non-communicable diseases, frequently ill individual due to communicable diseases and higher educational status of the households. Perceived poor quality of health care in terms of shortage of drugs and supplies, lack of trust of the community on commitment of CBHI administrators, lack of transparency of CBHI scheme management were the reasons listed by the community for non-enrollment. Wide-range awareness creation activities through locally available social marketing strategies are beneficial. Capacity building for CBHI administrators and trust building among communities through transparent management should be given attention. In addition, the quality of care being given in public health facilities should be improved. We suggest further operational research to assess the readiness of health facilities to accommodate the patients’ expectation and the barriers in the study area.

**conclusion section in the abstract: The study found that lower community based health insurance enrollment status. Higher probability of CBHI enrollment among higher health care demanding population groups was observed. Poor perceived quality of health care, poor managerial support and lack of trust were found to be barriers for non-enrollment. Therefore, wide-range awareness creation strategies should be used to address adverse selection and poor knowledge. In addition, trust should be built among communities through transparent management. Furthermore, the quality of care being given in public health facilities should be improved to encourage the community to be enrolled in CBHI.

**Authors response for reviewer 2**

Reviewer #2: The authors investigated supply-side and adverse selection factors in the enrollment in community-based health insurance (CBHI) in selected zones in Ethiopia, Africa. The research topic/area is of interest towards providing good health care coverage to the sampled citizens. However, the manuscript cannot be accepted as it currently stands because of its quality and a number of issues which should be addressed by the authors. I recommend a Major review and re-submission subject to the corrections of the under-listed issues.

(1) The title of the manuscript is too long and clumsy. Authors should among other titles, consider: Determining the factors influencing the selection and enrollment in community-based health insurance scheme in Ethiopia.

**Response**: **Title** **editing effected accordingly**

Factors affecting enrollment status of households for community based health insurance in a resource-limited peripheral area in Southern Ethiopia.

(2) The study appears to lack novelty and have few innovations that could be readily pointed to. No new method(s)/technique(s) was introduced as authors just applied existing method(s) to their study without any creativity attached.
Authors should specifically mention their study's innovations and contributions that makes it unique and state-of-the-art.
The need for utilizing some methods including thematic analysis, narrative weaving, binary logistic regression etc should be briefly explained.

**Response:**

Here the concern of the reviewer is lack of novelty. This research is not basic research (type of research its purpose is innovation/ generating new knowledge) rather it is applied research which concentrates on identifying problems and finding solutions to the problems. Such type of study is very important for evidence based practice. In fact, there are published articles in this specific area of interest but three things should be taken in to consideration.

1. The study setting is quite different. In Ethiopian context, there are accessible and inaccessible areas for health services and information. Accordingly our research addresses the equity issue related to adequate information to intervene public health problem which is related to alternative financing mechanism.
2. Our study has exhaustively addressed supply side factors by qualitative method which usually is not addressed by many studies including the mentioned study in Ethiopia
3. Other studies published in this specific topic are out of Ethiopia and even their variable of interest is on willingness to enroll, not on enrollment status.

-Binary logistic regression is a type of analysis method which helps us to identify significant variables related to our outcome of interest with binary outcome.

Thematic analysis is “A method for identifying, analyzing and reporting patterns (themes) with in data”.

Ref: Braun & Clarke, 2006:79

-Kindly refer under data analysis section in the manuscript

(3) It is suggested that authors should include the sample questionnaires administered in the study as an addendum to the manuscript to enable the reviewers and other researchers have a better understanding.

**Response:** we have attached the questionnaire as supporting information

(4) The manuscript is not well structured and written in standard English language. There exist a lot of typos and grammatical errors that must be resolved. These affect the readability and understanding of the manuscript. For instance:

(i) inappropriate mixing, use and introduction of various upper case letters
(ii) poor use and outright omission of punctuation marks. Check the list of Authors' name, Authors' affiliations, Abstract
including wrong indexing (superscripts)
(iii) heath - instead of health
(iv) "The study instruments/tools for this study..."
(v) "health development army"
(vi) "The other reason for low reason..."

**Response:** Dear reviewer, we appreciate and thank you for your meticulous observation of the error we made in reporting.

We have revised the whole manuscript document for typographical and grammatical errors.

(5) Woreda; kebele: First appearance of these words on page 2 and 3 respectively does not indicate their meaning.

Woreda—equivalent with district in Ethiopia

Kebele----smallest administrative unit in Ethiopia

*Kindly the correction is effected

(6) What informed the authors' selection of the Segen area and South Omo Zones for this research study especially as it was also stated in the Discussion section that these areas are prone to political instability?

**Response:** These areas are politically instable as well as they are very remote zones of Southern region.

(7) Some of the statements in the manuscript are vague. For instance on page 3,
(i) "The administrative offices of the zones are located about 750kms south of Addis Ababa".
Authors should state what administrative offices are.
(ii) "But one of the district's population are semi-pastoralist and pastoralist" - which district?

**Response**: kindly resolved

(8) There are two Table 2 in the manuscript - kindly resolve.
Authors should also stick to just one of these words "Not Correct" or "Incorrect" as shown in Table 2 to aid understanding.

**Response** : kindly resolved

(9) The various Tables in the manuscript are not explained, referred to and properly linked to the statements in the body of the manuscript. Some of these Tables are simply counting, percentages or frequency related and not statistically rich enough. Some acronyms e.g. HH in Table 2 are not provided meanings.

**HH**…households

**Kindly resolved**

(10) Consider revising the captions of the Tables in the manuscript. They are too long and contains repeated phrases.

**Kindly resolved**

(11) The factors identified from the qualitative results and listed on page 14 were just presented as obtained from respondents without authors' input and explanations including their impacts and relating these findings to the current study.

**Response**: We really appreciated the reviewers concern regarding lack of synthesis for the quotes. Kindly, the reviewer can find the themes, synthesis and quotes for each synthesis in qualitative data presentation.

(12) Numbered references in the body of the manuscript should be in square brackets. Some references doesn't even have yaer of publication stated. The referencing style adopted by the authors is not consistent. Kindly check and adapt to Plosone reference style.
**Response**: Kindly resolved

**Response for reviewer 3**

Reviewer #3: The manuscript is technically sound and the methodology was done with rigor. The study investigates a critical aspect of access to health care that can significantly contribute to UHC. My main comment is the conclusion appears to be biased towards identifying adverse selection factors such as age, illness, education etc. On the other hand majority of non CBHI users indicated that lack of quality of service is the reason why they are not enrolled in the CBHI. I recommend the availability of quality services to be adequately reflected in the conclusion.

Response: kindly correction is effected

*Correction is effected as follows:

Conclusion section in the Manuscript: There was lower enrollment status of the households in community based health insurance (CBHI) scheme. We found that enrollment to community based health insurance was higher among people who had good knowledge, higher family size, presence of children whose age (≤18 years) and or elders (65+ years), presence of a household member with chronic non-communicable diseases, frequently ill individual due to communicable diseases and higher educational status of the households. Perceived poor quality of health care in terms of shortage of drugs and supplies, lack of trust of the community on commitment of CBHI administrators, lack of transparency of CBHI scheme management were the reasons listed by the community for non-enrollment. Wide-range awareness creation activities through locally available social marketing strategies are beneficial. Capacity building for CBHI administrators and trust building among communities through transparent management should be given attention. In addition, the quality of care being given in public health facilities should be improved. We suggest further operational research to assess the readiness of health facilities to accommodate the patients’ expectation and the barriers in the study area.

**conclusion section in the abstract: The study found that lower community based health insurance enrollment status. Higher probability of CBHI enrollment among higher health care demanding population groups was observed. Poor perceived quality of health care, poor managerial support and lack of trust were found to be barriers for non-enrollment. Therefore, wide-range awareness creation strategies should be used to address adverse selection and poor knowledge. In addition, trust should be built among communities through transparent management. Furthermore, the quality of care being given in public health facilities should be improved to encourage the community to be enrolled in CBHI.

**Response for Reviewer 4**

Reviewer #4: Universal health coverage is essential to ensure global access to health care. It is an important subject for research in resource poor settings. This study uses sound methodology to investigate possible factors affecting the uptake of CBHI. The authors have made a number of typographical and grammatical errors. They use terms not of English origin and have omitted giving meaning to these words. The discussion needs more work in drawing comparisons with other similar research to draw conclusions, make adequate recommendations and suggestions for further research. I have uploaded a copy of the manuscript with comments.

**Response:** Dear reviewer, we appreciate and thank you for your meticulous observation of the error we made in reporting.

We have revised the whole manuscript document for typographical and grammatical errors.

We have corrected all your concerns in the manuscript document…kindly indicated using track change.

Were the households used in the piloting process included in final data analysis?

Response: Not included in the final data analysis
